# Usefulness of a Multiparent Advanced Generation Intercross Population With a Greatly Reduced Mating Design for Genetic Studies in Winter Wheat

**DOI:** 10.3389/fpls.2018.01825

**Published:** 2018-12-06

**Authors:** Melanie Stadlmeier, Lorenz Hartl, Volker Mohler

**Affiliations:** ^1^Bavarian State Research Center for Agriculture, Institute for Crop Science and Plant Breeding, Freising, Germany; ^2^TUM School of Life Sciences Weihenstephan, Technical University of Munich, Freising, Germany

**Keywords:** multiparental population, SNP, RIL, linkage map, recombination, wheat

## Abstract

Multiparent advanced generation intercross (MAGIC) populations were recently developed to allow the high-resolution mapping of quantitative traits. We present a genetic linkage map of an elite but highly diverse eight-founder MAGIC population in common wheat (*Triticum aestivum* L.). Our MAGIC population is composed of 394 F_6:8_ recombinant inbred lines lacking significant signatures of population structure. The linkage map included 5435 SNP markers distributed over 2804 loci and spanning 5230 cM. The analysis of population parameters, including genetic structure, kinship, founder probabilities, and linkage disequilibrium and congruency to other maps indicated appropriate construction of both the population and the genetic map. It was shown that eight-founder MAGIC populations exhibit a greater number of loci and higher recombination rates, especially in the pericentromeric regions, compared to four-founder MAGIC, and biparental populations. In addition, our greatly simplified eight-parental MAGIC mating design with an additional eight-way intercross step was found to be equivalent to a MAGIC design with all 210 possible four-way crosses regarding the levels of missing founder assignments and the number of recombination events. Furthermore, the MAGIC population captured 71.7% of the allelic diversity available in the German wheat breeding gene pool. As a proof of principle, we demonstrated the application of the resource for quantitative trait loci mapping analyzing seedling resistance to powdery mildew. As wheat is a crop with many breeding objectives, this resource will allow scientists and breeders to carry out genetic studies for a wide range of breeder-relevant parameters in a single genetic background and reveal possible interactions between traits of economic importance.

## Introduction

In plant breeding, the detection of quantitative trait loci (QTL) is no longer limited by the availability of genetic marker information and genotyping throughput (Mammadov et al., 2012; Chen et al., 2014; He et al., 2014; Unterseer et al., 2014; Cui et al., 2017), but rather by the genetic material employed (Flint-Garcia et al., 2003; Zhu et al., 2008; Asimit and Zeggini, 2010; Gibson, 2012). In an attempt to counteract this fact, nested association mapping (NAM) and multiparent advanced generation intercross (MAGIC) populations were established. In crops, Mackay and Powell (2007) and Cavanagh et al. (2008) first discussed the latter population type. In MAGIC designs, multiple inbred founders are intercrossed several times in a well-defined order to combine the genetic material of all the founders in a single line (Cavanagh et al., 2008). This leads to highly diverse genotypes each with a unique mosaic of founder alleles. The higher number of parents and recombination events of a MAGIC population are clear advantages compared to a classical biparental population, while for both designs pedigree and genetic structure are well known. In association mapping (AM) panels, genetic diversity and recombination rates are higher than in MAGIC designs as these panels take advantage of a collection of diverse breeding lines. However, the main factor limiting AM studies is confounding due to population structure based on sampling effects (Flint-Garcia et al., 2003; Caldwell et al., 2006; Vilhjálmsson and Nordborg, 2013) which enhances the risk of detecting false positives (Ewens and Spielman, 2001; Dickson et al., 2010; Korte and Farlow, 2013). Therefore, MAGIC populations represent an intermediate to biparental crosses and diversity panels concerning substructure, allele diversity, the number of traits that can be investigated, resolution, and power (Rakshit et al., 2012; Pascual et al., 2016). To date, MAGIC populations were established in a wide range of various crops including rice (Bandillo et al., 2013), tomato (Pascual et al., 2015), fava bean (Sallam and Martsch, 2015), maize (Dell’Acqua et al., 2015), barley (Sannemann et al., 2015), and sorghum (Ongom and Ejeta, 2018). In wheat, the first described MAGIC population was based on four founder genotypes (Huang et al., 2012). Further wheat MAGIC resources were generated using eight parents by Mackay et al. (2014) and Sannemann et al. (2018). A 16 founder wheat MAGIC population was established in the UK including elite and historical varieties (Fradgley et al., 2017). It is expected that a higher number of parents and initial crosses will result in a better dissection of complex traits (Huang et al., 2012). However, this implies more time and higher costs for population creation. So far, no study has been published investigating a MAGIC population design that involved a greatly reduced number of overall crossings.

Through intensive genetic analysis, we evaluated the impact of a MAGIC mating design with a greatly reduced number of overall crossings. First, we explored the genetic structure of our Bavarian MAGIC wheat population (BMWpop) for the validation of the crossing procedure. Second, we constructed a genetic linkage map, which we evaluated through population parameters and congruency with other maps. For comparative analysis, we used two other wheat MAGIC maps (Cavanagh et al., 2013; Gardner et al., 2016), one biparental map (Geyer et al., 2017), and the IWGSC RefSeq v1.0 (International Wheat Genome Sequencing Consortium, 2018). Recombination fraction, linkage disequilibrium, and founder probabilities were used to address population analysis parameters. Third, as a proof of principle, we demonstrated the application of the resource for QTL mapping using the trait seedling resistance to powdery mildew. The described genetic material will be shared with the scientific community upon request. It will enable scientists and breeders to carry out genetic studies for a wide range of economically relevant winter wheat breeding traits in a single genetic background and to reveal possible interactions.

## Materials and Methods

### Plant Material and Population Development

**FIGURE 1:**
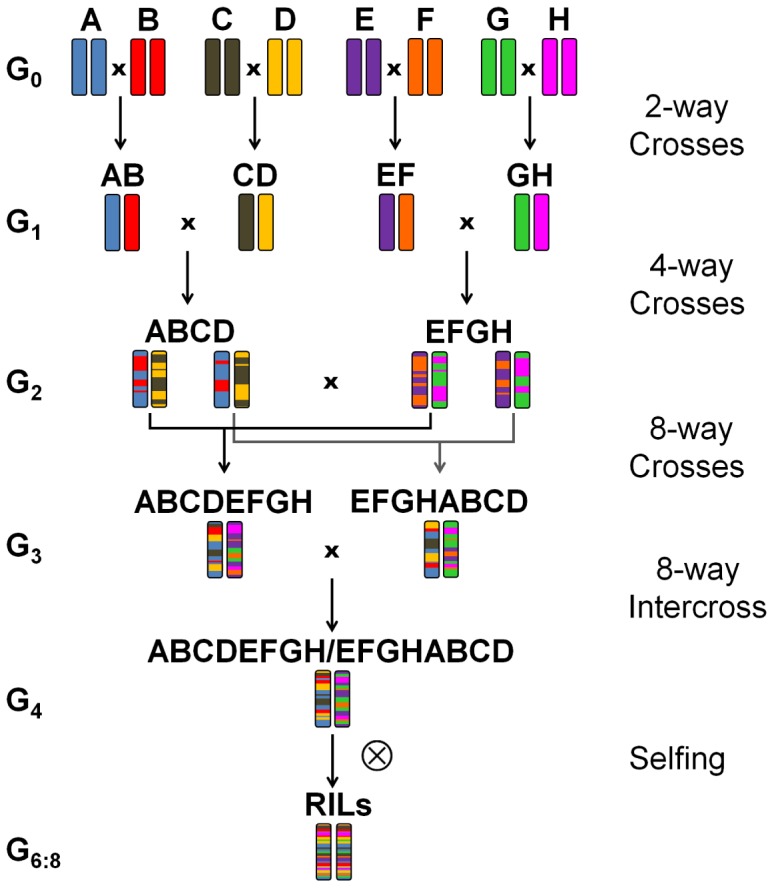
Crossing scheme based on Cavanagh et al. (2008) of the eight-founder BMWpop. **(A)** ‘Event’, **(B)** ‘Format’, **(C)** ‘BAYP4535’, **(D)** ‘Potenzial’, **(E)** ‘Ambition’, **(F)** ‘Bussard’, **(G)** ‘Firl3565’, **(H)** ‘Julius’.

Eight winter wheat lines ‘Event’ (A), ‘Format’ (B), ‘BAYP4535’ (C), ‘Potenzial’ (D), ‘Ambition’ (E), ‘Bussard’ (F), ‘Firl3565’ (G), and ‘Julius’ (H) were selected as founders of an eight-way MAGIC population based on the following criteria: (i) variation for disease resistance, quality, and agronomic traits, (ii) derivation from diverse breeding programs, and (iii) importance within the respective quality group (Table 1). All founders originated from wheat breeders in Germany except for the variety ‘Ambition’, which emerged from the Nordic Seed (Denmark) breeding program. To create F_1_ seed, one ear per plant was used in four two-way crosses (AB, CD, EF, and GH) (Figure 1). Out of each of the four two-way crosses, one randomly selected F_1_ seed was raised and two ears per plant were further mated to four-way crosses (ABCD and EFGH). Out of 32 available four-way crosses, sixteen independent plants were selected and crossed, to obtain eight F_1_ populations that involved four reciprocal cross combinations (ABCDEFGH and EFGHABCD). The four eight-way crosses were further hybridized with the four reciprocal eight-way crosses for establishing 16 eight-way intercross combinations (ABCDEFGH/EFGHABCD). The term ‘MAGIC group 1–16’, as used hereinafter, refers to these 16 subpopulations. The F_1_ seeds of BMWpop were progressed to the F_6_ generation via single seed descent followed by two generations of bulk propagation in the field. The crossing procedure started in June 2009, and was completed in December 2010 followed by 5 years it took to self the plants to F_6:8_ recombinant inbred lines (RILs).

**Table 1:** Classification of founder lines of the eight-parent Bavarian MAGIC winter wheat population.

Founder line	Quality			Resistance			Breeder	Registration period
					
	Group^c^	GPC^d^	Yield^e^	PM^f^	LR^f^	STB^f^		
Event^a^	E	6	7/5	3	4	6	Saatzucht Josef Breun	2009–2015
Format^a^	A	8	6/6	5	5	4	Saatzucht Schweiger	2007–2013
BAYP4535^b^	C	n.d.	7/7	2	2	4	LfL	Not released
Potenzial^a^	A	5	7/7	2	4	5	Deutsche Saatveredelung	2006–present
Ambition^a,b^	C	4	6/7	2	5	4	Nordic Seed	2003–2012
Bussard^a^	E	8	2/3	4	7	6	KWS Lochow	1990–present
Firl3565^b^	A	4	6/7	4	5	4	Saatzucht Firlbeck	Not released
Julius^a^	B	4	8/8	3	3	3	KWS Lochow	2008–present


*^*a*^Based on the descriptive variety list of the Federal Plant Variety Office 2009 (www.bundessortenamt.de).*

*^*b*^Based on the assessment of the Bavarian Research Center for Agriculture (LfL).*

*^*c*^Quality group (bread-making): E (excellent), A (high), B (medium), and C (low/other use).*

*^*d*^Grain protein content: 1 (low) – 9 (high).*

*^*e*^Grain yield without growth regulator and fungicide treatment/at high production intensity: 1 (low) – 9 (high).*

*^*f*^PM (Powdery mildew), LR (Leaf rust), STB (Septoria tritici blotch); rating score: 1 (no disease) – 9 (severe disease).*

*n.d., not determined.*

### DNA Extraction and Genotyping

Ten individual primary leaves of each one of the 394 F_6:8_ RILs and all eight founders were harvested, pooled and freeze-dried. Genomic DNA was extracted according to the procedure described by Plaschke et al. (1995). All lines including the parents repeated twice were genotyped using the 15K + 5K Infinium^®^ iSelect^®^ array containing 17267 single nucleotide polymorphism (SNP) markers provided as a service by the company TraitGenetics (Gatersleben, Germany). The array combines markers from the 90K iSelect array (Wang et al., 2014) and the 820K Axiom^®^ array (Winfield et al., 2016). The population was further genotyped with a functional PCR marker for powdery mildew resistance allele *Pm3a* (Tommasini et al., 2006) since the parent ‘BAYP4535’ is known to carry that gene.

### Marker Filtering

The filtering of the genotypic data was carried out with the synbreed package V0.12-6 (Wimmer et al., 2012) in R (R Core Team, 2017). First, all markers were excluded which were monomorphic for the population and for the founders. Further markers were removed based on the following conditions: (i) missing data ≥ 5%, (ii) minor allele frequency ≤ 5%, (iii) duplicated segregation pattern (marker with the lowest number of missing data was kept), (iv) missing and heterozygous data in founders and (v) different allele calls between the double-genotyped founders. Markers exhibiting segregation distortion were left in the data set, as segregation distortion does not affect recombination fractions (Kjær et al., 1995).

### Population Validation

The genotypic validation of BMWpop was based on a subset of 6717 markers (selection procedure see section ‘2.2 Marker Filtering’). The population structure was investigated with a principle coordinates analysis (PCoA) based on Roger’s distance matrix in R/ape V5.0 (Paradis et al., 2004). The kinship matrix K was calculated with the R package Genomic Association and Prediction Integrated Tool (GAPIT Tool 3.0) (Lipka et al., 2012) using the method of VanRaden (2008). Furthermore, we examined the proportion of heterozygote allele calls i.e., the combination of the heterozygosity of each plant and the segregation of the allele within the ten bulked plants of the F_6:8_ lines. The deviation of the observed segregation from the expected ratio of each marker was analyzed after setting all heterozygous data to missing. The multipoint probability of each locus that the observed genotype was inherited from one of the eight founders was calculated based on haplotype structure using the function ‘mpprob’ implemented in R/mpMap V2.0.2 (Huang and George, 2011). The threshold to determine the parental origin of an allele was set to 0.7. This threshold allowed us to compare the proportion of missing founder probabilities and the number of recombination events with other studies. The number of recombination events in the RILs of BMWpop was compared to the recombination events in the eight-founder MAGIC population NIAB 2015 (Gardner et al., 2016), the four-founder MAGIC design 9kMAGIC (Cavanagh et al., 2013), and the biparental backcross population L19 BC1 (Geyer et al., 2017). Information about the population size and type, the generation of the lines, and the genotyping platform used of the populations mentioned above is given in Table 2. Recombination events of RILs in all MAGIC populations were recognized by the change of the origin of parental alleles along the chromosome, whereas in the L19 BC1 lines the change of allele calls from heterozygote to homozygote along the chromosome and vice versa was assessed. Genome-wide recombination counts were available for 9kMAGIC (Huang et al., 2012), whereas for NIAB 2015 data were only published for chromosome 3B. The allelic diversity captured in BMWpop was compared to a panel of 524 common wheat breeding lines provided by six breeding companies (Geyer et al., 2016). This genetic library represents well the germplasm of the German wheat breeding pool and was genotyped with the Illumina^®^ Infinium^®^ 15 k SNP array representing a subset of markers of the 15K + 5K Infinium^®^ iSelect^®^ array.

**Table 2:** Description of BMWpop and reference populations and linkage map summary statistics.

	BMWpop	NIAB 2015^a^	9kMAGIC^b^	L19 BC1^c^
**Population type**	**8-founder MAGIC**	**8-founder MAGIC**	**4-founder MAGIC**	**Biparental**
Generation	F6:8	F4:5	F6	BC1
Population size	394	643	1440	230
SNP array	20k	80k	9k	20k
**Total marker number**	**5436**	**18601**	**4300**	**2100**
Marker number A genome	2185	6832	2055	967
Marker number B genome	2603	9388	1944	906
Marker number D genome	648	2381	301	227
Min number/chromosome	35	80	14	9
Max number/chromosome	483	2327	451	231
**Total chromosome length**	**5230**	**5405**	**3522**	**2823**
Chromosome length A genome	2012	2009	1420	1283
Chromosome length B genome	2070	2108	1405	1109
Chromosome length D genome	1149	1287	697	579
Min chromosome length	87	126	44	30
Max chromosome length	390	386	300	219
**Total loci number**	**2804**	**4578**	**1813**	**1920**
Loci A genome	1126	1892	798	874
Loci B genome	1327	2160	878	838
Loci D genome	351	526	137	208
Min loci/chromosome	25	39	11	9
Max loci/chromosome	248	452	202	209
**Mean recombination event/RIL**	73	–^d^	37	53
Range recombination event/RIL	49–100	–^d^	1–80	23–126


*^*a*^Mackay et al., 2014; Gardner et al., 2016.*

*^*b*^Huang et al., 2012; Cavanagh et al., 2013.*

*^*c*^Geyer et al., 2017.*

*^*d*^No whole genome data available, only for chromosome 3B.*

### Map Construction

The construction of the genetic map was based on the two R packages mpMap V2.0.2 (Huang and George, 2011) and mpMap2 V0.0.3^1^^,^^2^ . Unless otherwise stated, the functions used were from the mpMap package. Heterozygous calls were set to missing. A recombination fraction (rf) matrix between all pairs of markers was calculated using the function ‘estimateRF’ in the package mpMap2 at default settings. The estimation of the rf matrix was based on identical by descent probabilities given in Broman (2005). Markers were grouped to 200 linkage groups using ‘mpgroup’ function. The linkage groups were assigned to chromosomes based on the information of published maps (Cavanagh et al., 2013; Wang et al., 2014; Marcotuli et al., 2017). Within linkage groups, markers were ordered in two steps using two-point ordering: First, an overall path order was constructed to minimize total map length using the ‘mporder’ function; second, fine ordering was performed based on the overall path order using the ‘orderCross’ function in mpMap2. All linkage groups belonging to the same chromosome were merged based on the consensus map of Cavanagh et al. (2013). Finally, the validity of the marker order over the whole chromosome was examined using the R/qtl ripple function implemented in the ‘mporder’ function. Genetic map distances were calculated using Haldane mapping function using ‘computemap’.

### Map Validation

Chromosome length and number of markers and loci were compared to the wheat genetic maps of NIAB 2015, 9kMAGIC, and L19 BC1 populations. The congruency of marker order was assessed in relation to the three above-mentioned genetic maps and to the physical marker positions of IWGSC RefSeq v1.0 (International Wheat Genome Sequencing Consortium, 2018) employing a set of markers common between BMWpop and the respective other population.

The rf heatmaps (R/lattice V0.20-35; Sarkar, 2008) were used to visualize marker order. Linkage disequilibrium (LD) was estimated as the squared correlation coefficient between markers using R/genetics V1.3.8.1 (Warnes et al., 2013). The extent of LD decay was analyzed similar to Breseghello and Sorrells (2006): a population-specific *r*^2^ value was determined at the 95th percentile of the square root transformed *r*^2^ value distribution for unlinked markers. *r*^2^ values above this threshold were expected to be caused by genetic linkage. A least square regression (loess) curve was fitted to the LD decay estimation using a smoothing span parameter of 0.10 (R/stats V3.4.3, R Core Team, 2017).

### QTL Mapping

Two independent trials were conducted for powdery mildew (PM) seedling resistance in the glasshouse under controlled conditions with a mean relative humidity of 67%, a temperature of 18°C, and 16 h of light per day. For each experiment, the population and the eight founders were sown in multi pot plates in a randomized complete block design with two replicates each. After the primary leaf emerged up to a length of 5 cm, the plants were inoculated with a *B. graminis tritici* isolate mixture by evenly shaking conidia of heavily infected wheat seedlings above the trial. The isolate mixture consisted of two isolates with virulence to the resistance genes *Pm2, Pm4b, Pm5a, Pm6*, and *Pm8* (Mohler et al., 2013). Disease reaction was scored from 1 (no symptoms) to 9 (susceptible) 2 weeks after inoculation. The assessment was based on ten plants per genotype and replicate.

Phenotypic data were adjusted in R/lme4 V1.1-14 (Bates et al., 2014) based on the following model:

(1) $$y_{ijk}=\mu +g_{i}+l_{j}+gl_{ij}+r_{kj}+e_{ijk}$$

where *y_ijk_* is the trait observation, μ is the overall mean, *g_i_* is the fixed effect of genotype *i, l_j_* is the random effect of the trial *j, gl_ij_* is the random interaction effect of genotype *i* with trial *j, r_kj_* is the random effect of replication *k* nested within trial *j*, and *e_ijk_* is the random residual error. To obtain variance components, the genotype was fitted as random. Heritability was estimated on a progeny mean basis according to Hallauer and Miranda (1981).

The QTL mapping was carried out using the mpMap package V2.0.2. Simple interval mapping was based on founder probabilities computed as mentioned above (2.4 Population validation). The function ‘mpIM’ was used for mapping. QTL were detected at a genome-wide significance threshold of α < 0.001. The threshold was derived from an empirically null distribution with 1000 simulation runs similar to Churchill and Doerge (1994). All detected QTL of the base model were simultaneously fitted in a full model using the function ‘fit’. From this model fit, only QTL were kept with a p-value < 0.05 and the full model was fitted again to obtain additive founder effects relative to Julius and the phenotypic variance explained (*R*^2^) by individual QTL. The QTL support interval (S.I.) was defined as the map distance in cM surrounding a QTL peak at a -log10(p) drop of ±1.0. The designation of QTL followed the recommended rules for gene symbolization in wheat (McIntosh et al., 2013).

### Data and Material Availability

All raw genotypic data and the complete pedigree traceable to two-way crosses are publicly available at http://doi.org/10.14459/2018mp1435172. Seed of all 394 RILs of BMWpop and the eight founders are available for non-commercial use upon request from the Bavarian State Research Center for Agriculture (Freising, Germany).

## Results

### Construction of the MAGIC Population

The crossing design of the MAGIC population was greatly simplified compared to the maximum number of possible crosses (Mackay et al., 2014); however, it involved an additional eight-way intercross step. Any developed line can be sourced to one of the 16 eight-way intercrosses (MAGIC group 1-16). The RILs built up MAGIC groups of 13 to 58 lines with an average size of 32 lines. Altogether 972 seeds, each representing a unique genotype, were generated during MAGIC crossing procedure. Five hundred sixteen lines were advanced to F_6:8_ generation and a number of 394 lines were selected based on sufficient seed availability for field trials and suitability for experiments in the agricultural environment.

### Genotypic Data Analysis

**FIGURE 2:**
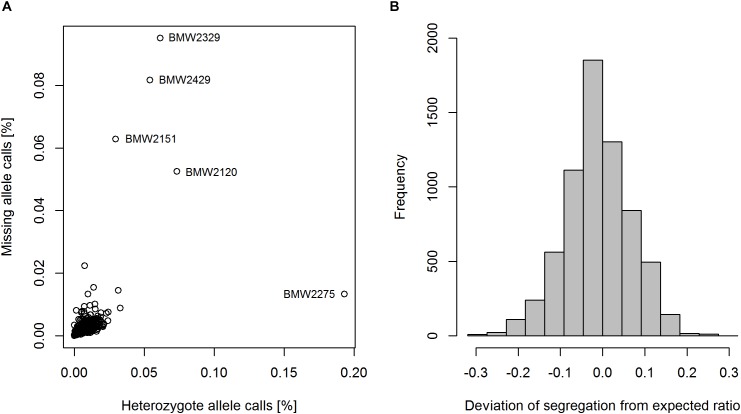
Heterozygosity and proportion of missing marker data of RILs **(A)**, and the segregation ratio of major and minor allele per marker **(B)**.

Of the 17267 SNP markers available on the 15K + 5K Infinium^®^ iSelect^®^ array, 11426 markers (66.2%) were segregating in the population. After marker filtering 6738 SNPs (58.8%) remained. Since founder lines were genotyped twice, a genotyping error rate of 0.1% could be observed. Twenty-two markers were found to be polymorphic in the population but not in the founders. These markers were also excluded from the data set, and a total number of 6716 codominant SNP markers and one dominant PCR marker remained for further analysis. The percentage of missing marker data per line was in the range of 0.0–9.5%, with an overall mean of 0.3% (Figure 2A). The four lines ‘BMW2329’, ‘BMW2429’, ‘BMW2151’, and ‘BMW2120’ showed the highest proportion of missing allele calls (Figure 2A). The mean proportion of heterozygote allele calls of the bulk of ten plants per MAGIC RIL was 0.8%. RIL ‘BMW2275’ showed an unexpected high level of heterozygote allele calls of 19.3%, but also three of the four above-mentioned lines had higher proportions of heterozygote calls (Figure 2A). The deviation of the observed segregation from the expected ratio followed a normal distribution with a mean of -0.01 (Figure 2B). The range of the deviation was from -0.32 to 0.26. A total of 71.7% of markers segregating in a diversity panel described by Geyer et al. (2016) were also polymorphic in BMWpop.

### Structure of the MAGIC Population

**FIGURE 3:**
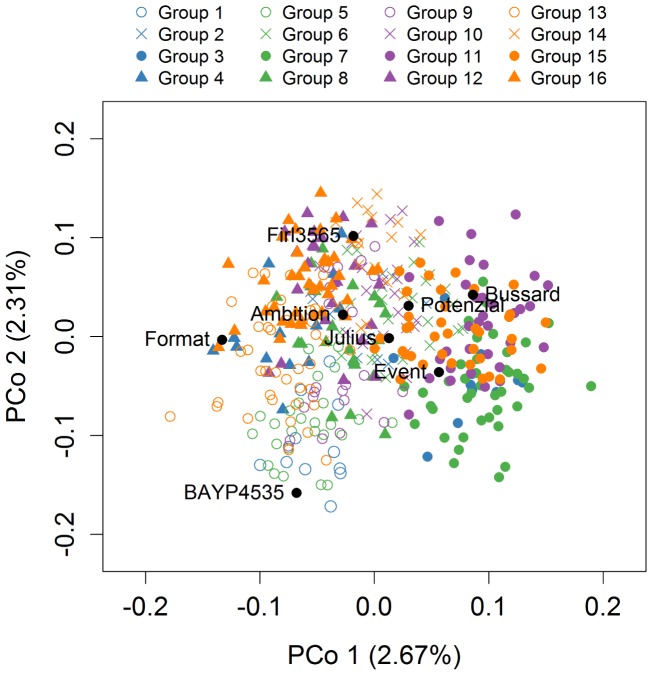
Population structure based on 6717 markers. Genotypes belonging to one of the 16 MAGIC groups are distinguished by color and symbol. The color represents the female crossing partner and the symbol represents the male crossing partner in the 8-way × 8-way crossing step.

**FIGURE 4:**
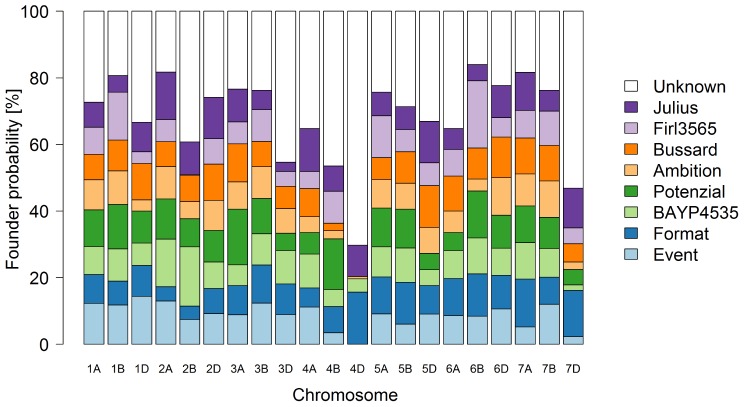
Genome-wide founder representation in the 394 eight-parent MAGIC RILs.

The first two principle coordinates of the PCoA accounted for 2.7 and 2.3% of the molecular variation, respectively (Figure 3). Individuals of MAGIC groups with the same paternal crossing partner in the 8-way × 8-way cross showed a weak clustering. The kinship showed that all lines were relatives and that there was no kinship structure present although lines built up some more related groups (Supplementary Figure S1). Thus, analyses revealed a mild population structure. Average congruency of the marker pattern between all lines was 62.8%. The range of the similarity of the marker pattern of two lines was from 52.2 to 97.6%. The genome-wide founder contributions in BMWpop were analyzed with haplotype probabilities using a threshold of 0.7. Based on the threshold setting, different proportions of founder probabilities per chromosome remained unknown (Figure 4). The average amount of missing founder contribution over the whole genome was 31.6%, ranging from 16.0% on chromosome 6B to 70.3% on chromosome 4D with average values of 26.0, 28.2, and 40.5% for the A, B, and D genomes, respectively. Generally, all eight parental genomes contributed to all chromosomes except for chromosome 4D. Based on an eight-founder crossing scheme as used in this study, each founder genome is expected to be represented by 12.5% in the population. In BMWpop, the average founder contribution varied from 7.0 to 9.6%. Generally, for the 18 chromosomes with higher explained contributions (Figure 4), the probability explained by the eight founders was quite equally distributed except for chromosome 2B. Here, the parent ‘Firl3565’ was underrepresented with 0.1%, whereas ‘BAYP4535’ was overrepresented with 17.8%.

### Genetic Map Construction

In total, 5436 markers were assigned to the 21 wheat chromosomes. Summary statistics are shown in Table 2. The complete genetic map of BMWpop is presented in Supplementary Table S1. The majority of markers of 40.2 and 47.9% were mapped to the A and B genomes, respectively. Only 11.9% of markers were located on the D genome. The number of markers per chromosome varied between 35 on chromosome 4D and 483 on chromosome 5B. The total linkage map spanned 5230 cM. The A and B genomes contributed equally to the map length with 38.4 and 39.6%, respectively, whereas the D genome had only a share of 22.0%. The individual chromosome length ranged from 87.0 cM for chromosome 4D to 389.6 cM for chromosome 7A. Average chromosome length was 287.4 cM, 295.7 cM, and 164.1 cM for the A, B, and D genomes, respectively. More than half of the mapped markers (2804) represented unique sites across the genome (A genome: 40.2%; B genome: 47.3%; D genome: 12.5%). The number of loci per chromosome was between 25 on chromosome 4D and 248 on chromosome 3B. The average locus distance on the A, B, and D genome was 1.8, 1.6, and 3.3 cM, respectively. A major gap of 53.9 cM was present in the pericentromeric region of chromosome 3A (Supplementary Figure S2, Chromosome 3A). In this region, no markers could be mapped because all markers associated with this region were monomorphic between the eight founder lines.

### Map Comparisons

Table 2 summarizes the comparison of BMWpop linkage map to three other maps. The eight-founder MAGIC map NIAB 2015 included 3.4× higher number of markers, whereas the maps of the four-founder 9kMAGIC population and the biparental L19 BC1 progeny had 0.8 and 0.4 as many markers, respectively. However, when considering the number of loci, the difference between NIAB 2015 and BMWpop was reduced to 1.6×. The two other maps showed 0.6 (9kMAGIC) and 0.7 (L19 BC1) as many loci. Both eight-founder MAGIC maps showed a similar total map length. The 9kMAGIC and L19 BC1 maps were 0.3× and 0.5× shorter, respectively. With respect to the sub-genome level, a similar trend for all investigated parameters was observed (Table 2).

In general, the marker order compared to the other three genetic maps, especially NIAB 2015, and the IWGSC RefSeq v1.0, was similar (Supplementary Figure S2). In some instances, local marker order was inverted compared to NIAB 2015 (Supplementary Figure S2, Chromosome 2B, panel B). Comparison of the D genome to 9kMAGIC and L19 BC1 was challenging due to an overall low marker number which limited the number of common markers required for the analysis (Supplementary Figure S2, Chromosome 3D, panel C,D). The comparison with the physical map clearly indicated lower recombination rates in the centromeric region of all chromosomes (Supplementary Figure S2, Chromosome 2A, panel A) except for chromosomes 3B, 5B, and 6B. In the three MAGIC populations, a similar coverage of marker loci was observed near the centromere (Supplementary Figure S2, Chromosome 2A, panel B,C), compared to a strong reduction in L19 BC1 (Supplementary Figure S2, Chromosome 2A, panel D). Further examination of marker order had the following outcomes: First, chromosome 6B showed low recombination rates in the region between 74.4 cM and 103.0 cM. This corresponds to approximately half of the length of the comparable region in NIAB 2015 that ranged from 101.1 cM to 167.8 cM (Supplementary Figure S2, Chromosome 6B, panel B). Second, there was an inversion on the long arm of chromosome 5D compared to NIAB 2015 (Supplementary Figure S2, Chromosome 5D, panel B), but not compared to the physical position and the consensus map of Wang et al. (2014) (Supplementary Figure S3). The average number of recombination events per RIL of BMWpop was 73.0, with values ranging from 49 to 100 (Table 2). There were 2x as many recombination events per line compared to four-founder MAGIC genotypes but just 1.4× as many compared to L19 BC1. For all populations except for the biparental one, average experimental recombination events lagged far behind simulated ones based on a wheat consensus map (Somers et al., 2004) with a total length of ∼2500 cM (Huang et al., 2012). BMWpop, 9kMAGIC, and L19 BC1 showed 71.6, 48.1, and 103.9% of the simulated average recombination number, respectively.

### Linkage Map Validation

The overall heatmap of the recombination fraction matrix (Supplementary Figure S4) showed a clear diagonal. The analysis of individual chromosomes reflected the signatures for correctly mapped A, B, and D genomes (Supplementary Figure S5; Gardner et al., 2016). The A and B genome chromosomes showed a distinct diagonal with small-sized tightly linked blocks (Supplementary Figure S5, Chromosome 5, panel A). In some cases, the markers around the centromeric region were all closely linked leading to bigger-sized blocks (Supplementary Figure S5, Chromosome 7, panel A). An extreme case of this was seen for chromosome 6B (Supplementary Figure S5, Chromosome 6, panel B). This result confirmed the already described truncated map length around the centromeric region of this chromosome (see “Map Comparison”). The rf heatmaps of the D genome chromosomes (Supplementary Figure S5, Chromosome 7, panel C) showed a limited number of blocks.

Plots of LD showed a clear pattern of intra-chromosomal LD decay in BMWpop for the genome and for the individual chromosomes (Supplementary Figures S6, S7). The mean LD for the genome decreased to *r*^2^ < 0.2 within 9.3 cM. Individual chromosome LD decay to *r*^2^ < 0.2 ranged from 3.5 cM (chromosome 6D) to 18.5 cM (chromosome 7D). To consider LD between loci due to genetic linkage the *r*^2^ critical population-specific threshold was 0.017. Thus for the genome, markers were assumed to be genetically linked within a distance of 68.6 cM.

### Powdery Mildew QTL

The repeatability of PM trials was 81.3 and 81.9% with a correlation between the trials of 0.8 (Supplementary Table S2). The residuals of the adjusted means followed a normal distribution (Supplementary Figure S8A), a prerequisite for appropriate QTL mapping. The progeny mean was not significantly different (*p* < 0.05) from the parental mean (Supplementary Table S2). The adjusted means (Supplementary Table S3) did not follow a normal distribution but rather showed a bimodal distribution (Supplementary Figure S8B). The heritability estimate was high with 93.0% (Supplementary Table S2). Five QTL were detected in simple interval mapping explaining 72.5% of the total phenotypic variance (Table 3). The individual *R*^2^ values ranged from 4.5 to 34.1%. The support interval for all QTL was between 0 and 9 cM. The strongest QTL *QPm.lfl-1A* explained 34.1% of the phenotypic variance and coincided with the functional marker for the *Pm3a* gene. The resistance allele was inherited from ‘BAYP4535’, which is the only founder carrying this gene, and reduced disease severity by 3.6 grades on a scale of 1–9. The QTL on chromosomes 6B and 7A had also high -log(p) values and accounted for 17.4 and 18.3%, respectively, of the variation for PM. The alleles reducing disease severity at *QPm.lfl-6B* and *QPm.lfl-7A* were contributed by ‘Event’ and ‘Ambition’, respectively. The QTL *QPm.lfl-1B* explained 4.5% of the phenotypic variance with all founder effects being negative relative to the parent Julius, whereas four parental lines decreased disease severity for *QPm.lfl-4A*.

**Table 3:** QTL for seedling resistance to powdery mildew (PM) in BMWpop.

Trait	QTL	Chr.	Pos. (cM)	S.I. (cM)	*P*-value	*R*^2^∗^^	Eff(A)	Eff(B)	Eff(C)	Eff(D)	Eff(E)	Eff(F)	Eff(G)
PM [1–9]						**72.5**							
	*QPm.lfl-1A*	1A	3	3–3	0.0E+00	34.1	0.1	-3.6	0.1	0.3	0.3	0.5	0.8
	*QPm.lfl-1B*	1B	142	138–146	4.1E-05	4.5	-1.5	-1.8	-1.4	-1.2	-2.4	-1.2	-1.8
	*QPm.lfl-4A*	4A	162	160–169	1.2E-08	6.6	-0.1	-1.2	-0.6	0.5	0.0	-0.9	0.1
	*QPm.lfl-6B*	6B	177	177–177	0.0E+00	17.4	-3.2	0.5	0.0	0.1	0.2	0.3	0.2
	*QPm.lfl-7A*	7A	379	375–380	0.0E+00	18.3	-0.3	0.2	-2.8	0.0	0.1	-0.1	-0.3


*Chromosome (Chr.), position (Pos.), support interval (S.I.), *p*-value, proportion of phenotypic variance explained (*R*^*2*^), and additive effects (Eff) of the founders Event (A), BAYP4535 (B), Ambition (C), Firl3565 (D), Format (E), Potenzial (F), and Bussard (G) relative to Julius are presented.*

*^∗^The explained phenotypic variance of the model fitting all detected QTL simultaneously is presented in bold above the individual *R*^*2*^ values for each QTL.*

## Discussion

A full eight-founder MAGIC crossing design is built up of 28 two-way crosses, 210 four-way crosses, and 315 eight-way crosses (Mackay et al., 2014). Starting with just four two-way crosses, 32 four-way crosses and eight eight-way crosses our mating design was greatly reduced but it involved sixteen additional eight-way intercrosses to compensate for the low number of overall crosses. We evaluated possible effects of this crossing design on population structure, number of recombination events, genetic map construction, founder probabilities, and QTL mapping.

Our greatly simplified MAGIC design and the small population size of 394 RILs did not cause a considerable structure in the population as indicated by population structure and kinship analyses (Figure 1 and Supplementary Figure S1). This notwithstanding, we observed a negligible impact of the male eight-way intercrossing partner. Possible biased maternal effects (Donohue, 2009; Roach and Wulff, 1987) cannot be ruled out as in our simplified crossing design the parent Event was the ultimate female parent of all 394 RILs. Another parameter that could have been affected is the number of recombination events. Based on a simulation model, the number of recombination events per RIL in a population is expected to increase with the number of founders and the number of crosses involved (Huang et al., 2012; Ladejobi et al., 2016). The average number of 73 recombination events per line in BMWpop compared to 37 in a four-founder MAGIC population (Table 2) supported the assumption of the simulation model. Despite this, the experimentally derived number of recombination events lagged for both MAGIC populations behind the simulated ones, possibly due to partially informative SNPs and missing founder assignments. This drawback of MAGIC designs was already described in other studies that used mating designs with a higher number of crosses (Huang et al., 2012; Sannemann et al., 2015; Gardner et al., 2016). A similar number of 1.6 and 1.8 recombination events per line per Morgan was detected on chromosome 3B for NIAB 2015 (Gardner et al., 2016) and BMWpop, respectively. Despite this observation, which is limited to a single chromosome, it could be still possible that in a simplified MAGIC mating design a lower number of unique recombinations is available. A simplified MAGIC design could also have led to lower accuracy in estimation of the rf matrix, which in turn would have adversely affected genetic map construction. Nevertheless, of 6717 markers passing quality control, 5436 markers were mapped to the 21 wheat chromosomes. The rf heatmaps (Supplementary Figures S4, S5), and the agreement of BMWpop map to other genetic maps and the IWGSC RefSeq v1.0 (Supplementary Figure S2) supported the appropriateness of marker ordering. Of the 1281 unmapped markers, the largest share of 49.3 and 36.0% was rejected due to too many missing values (>20%) in the rf matrix and marker ordering, respectively. The trivialization of both criteria resulted in incorrect marker order and artificially increased map length. Therefore, to ensure accurate mapping in BMWpop, strict quality control of the rf matrix and extensive manual curation during the mapping process was carried out. Similar conclusions were reported by Gardner et al. (2016) who used an almost fully realized mating design for MAGIC population creation. A valid genetic map and a sufficient number of recombinations are prerequisites for the determination of the parental origin of an allele. Increasing the number of crosses and progenies in a MAGIC population would increase the possible number of recombination events and could reduce the amount of missing founder assignments. However, the genome-wide proportion of missing haplotype data of our greatly reduced mating design compared to the mating design of NIAB 2015 with 643 RILs was similar with 17.3 and 18.6%, respectively. Therefore, it appears that a higher number of crosses will not enhance the proportion of alleles assigned to parental origin. Since founder assignments are based on haplotypes, increasing the number of unique founder haplotypes would considerably improve founder assignment. This aim may be achieved for example by using a higher number of SNP markers, including multiallelic marker systems, or new approaches to construct the haplotypes (Gabriel et al., 2002; Pook et al., 2018). Additionally, to keep the error rate low the threshold to determine the parental origin of an allele needs to be set in the upper range strengthening missing founder assignments. Beside technical issues, there could be also genetic reasons for missing founder probabilities such as a narrow genetic distance of the founder lines. Simulation studies showed that for randomly selected founders in an eight-founder MAGIC population 73.7% of the loci were polymorphic (Ladejobi et al., 2016). This proportion could be increased to 91.6% if the founders were selected based on a maximized number of segregating alleles. The captured allelic diversity of 71.7% in BMWpop indicated that our founders were selected randomly in terms of allelic diversity and a higher degree of polymorphism may have been possible by choosing other parents. The monomorphic region on chromosome 3A of BMWpop supported this finding as lines being polymorphic in this region are available in a German wheat breeding panel (Geyer et al., 2016; data not shown). Finally, the above-mentioned limitations could have touched the dissection of the genetic architecture of seedling resistance to powdery mildew. However, limitations of QTL analysis could not be observed in this study. Three major and two minor QTL with a total explained phenotypic variance of 72.5% were identified (Table 3). Additionally, the small support intervals of 0–9 cM obtained provided further evidence for appropriate QTL mapping. The successful genetic analysis of a complex trait demonstrated that our genetic material employed is useful for further gene mapping studies. As MAGIC designs combine the genetic material of several founders, it is possible to investigate multiple traits simultaneously which is usually limited in biparental populations particularly when adapted germplasm is used. Our future work will show whether the genetic analysis of a broad range of traits reveal, from a breeder’s perspective, desirable or undesirable genetic relationships which are expected to be described with great accuracy and reliability as suggested by our QTL analysis.

## Conclusion

It appears that a greatly reduced MAGIC mating design including an additional eight-way intercross step is equivalent to an almost full design. This refers to the total number of recombination events, the ability to construct an appropriate genetic map, and the proportion of missing founder assignments. Furthermore, we could show that QTL mapping for seedling resistance to powdery mildew was successful in the background of this simplified eight-founder MAGIC design. These findings are of great importance for researchers and scientists working on multiparental populations as the effort of developing such a population can be significantly reduced. The eight-founder BMWpop and its genetic linkage map is a valuable genetic resource that could allow scientists and breeders to carry out genetic studies for a wide range of breeder-relevant parameters in a single genetic background.

## Author Contributions

VM and LH conceived the study. MS conducted the experiments and analyzed the data. VM supervised the research. MS and VM drafted the manuscript. All authors read, edited, and approved the final manuscript.

## Conflict of Interest Statement

The authors declare that the research was conducted in the absence of any commercial or financial relationships that could be construed as a potential conflict of interest.

## Footnotes

1 https://github.com/rohan-shah/mpMap2Paper

2 https://github.com/rohan-shah/mpMap2
