# Supplementary material for: Usefulness of a Multiparent Advanced Generation Intercross Population With a Greatly Reduced Mating Design for Genetic Studies in Winter Wheat
Source: Front Plant Sci. 2018 Dec 6;9:1825. doi: 10.3389/fpls.2018.01825 (PMC6291512; doi:10.3389/fpls.2018.01825)
Supplement: Supplementary file 1 [file Data_Sheet_1.pdf]

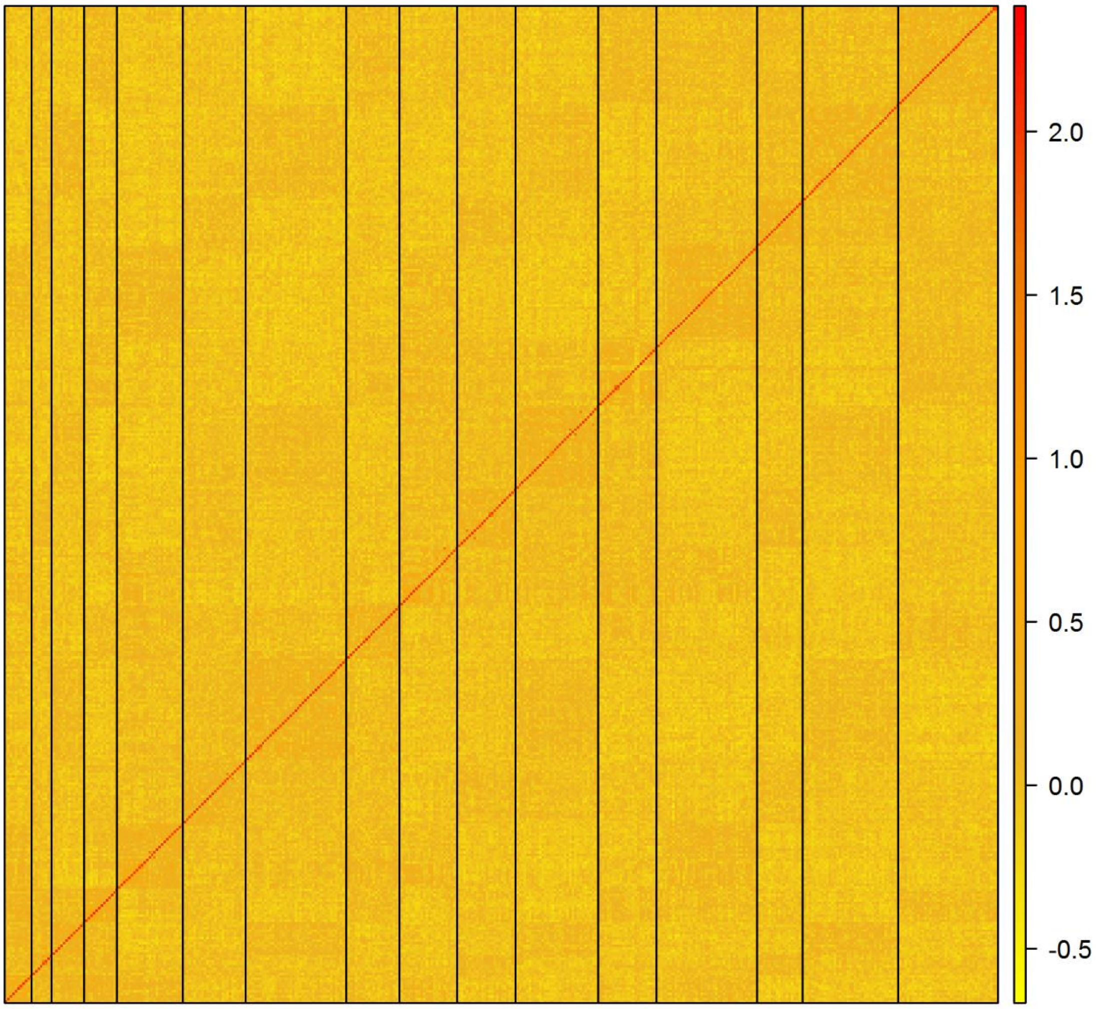

Figure S1: Kinship matrix. The lines are arranged according to the MAGIC groups starting with MAGIC 1 and ending with MAGIC 16. Black vertical lines indicate the border between two MAGIC groups. The more related two genotypes are, color turns from yellow (value '-0.7') over orange to red (value '2').
