# Supplementary material for: Usefulness of a Multiparent Advanced Generation Intercross Population With a Greatly Reduced Mating Design for Genetic Studies in Winter Wheat
Source: Front Plant Sci. 2018 Dec 6;9:1825. doi: 10.3389/fpls.2018.01825 (PMC6291512; doi:10.3389/fpls.2018.01825)
Supplement: Supplementary file 2 [file Data_Sheet_2.PDF]

Chromosome 1A

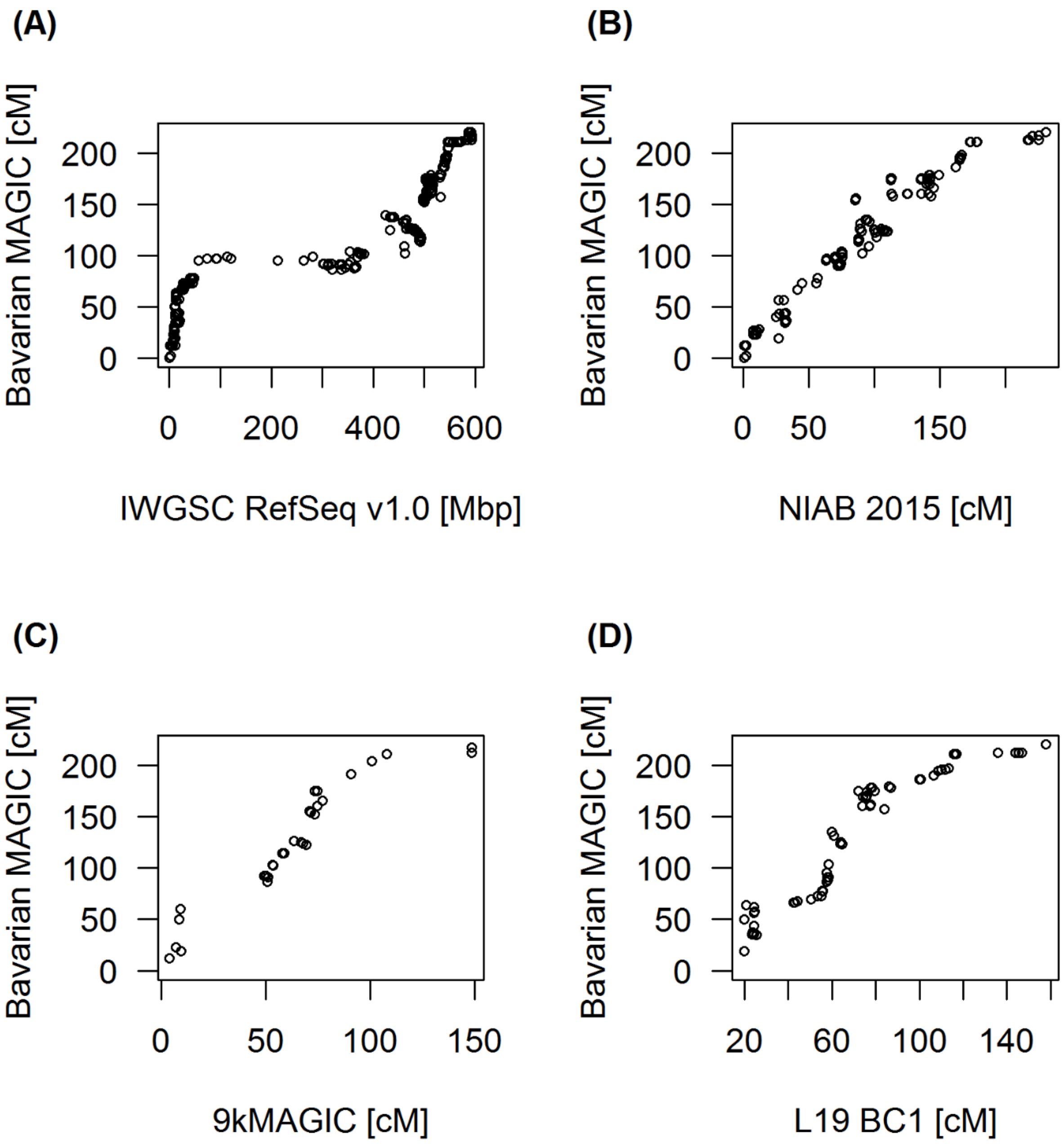

Chromosome 1B

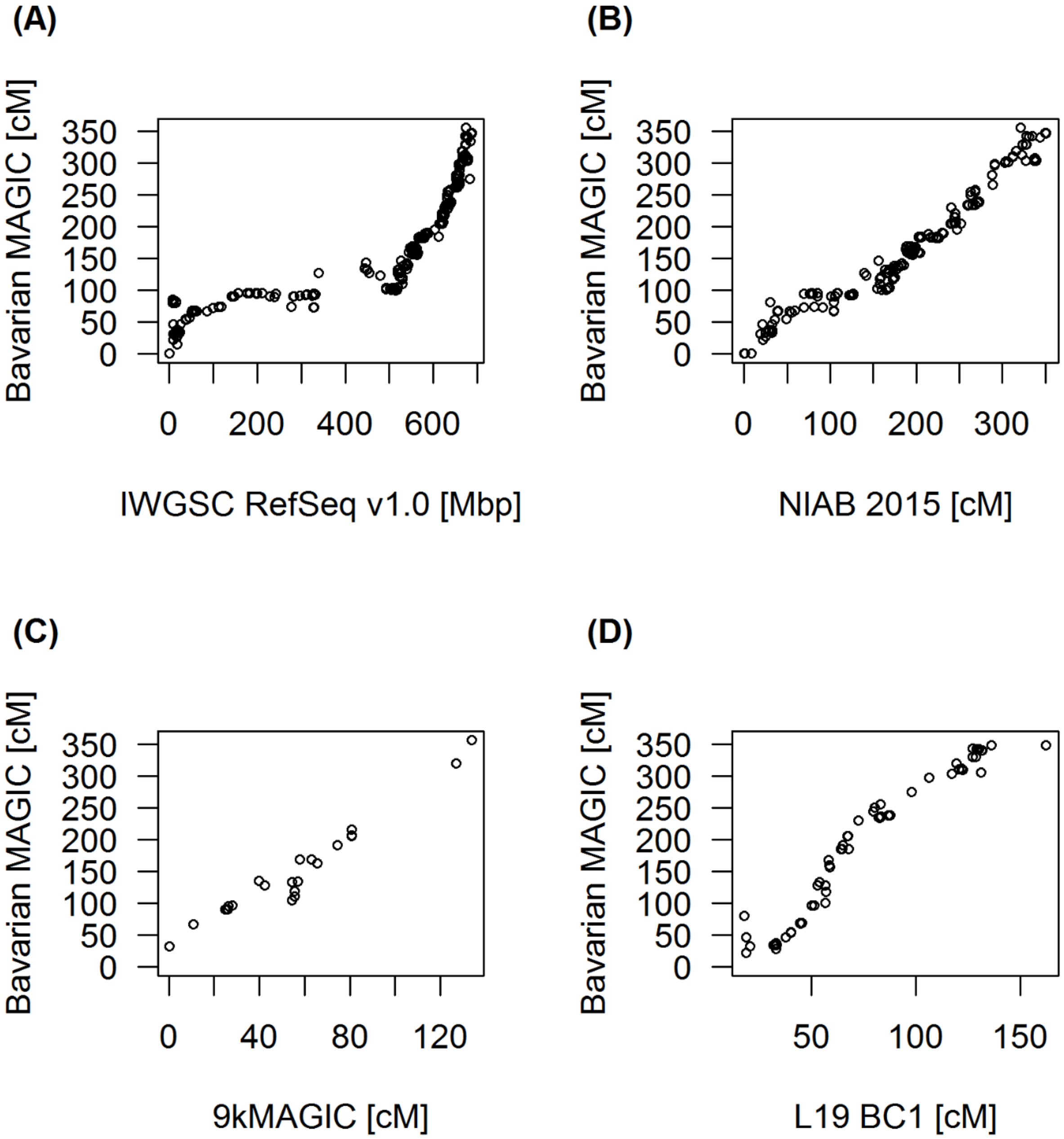

Chromosome 1D

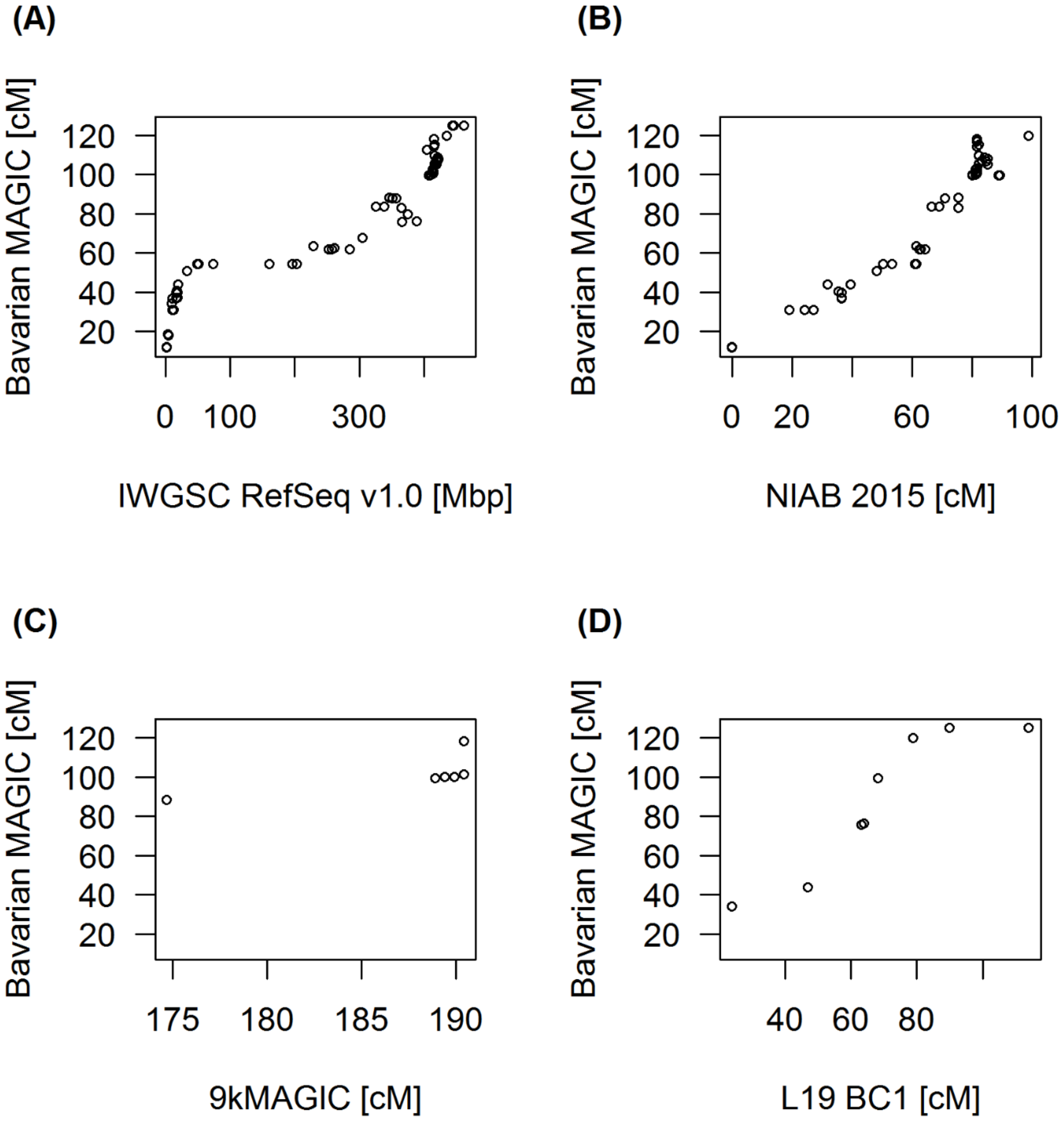

Chromosome 2A

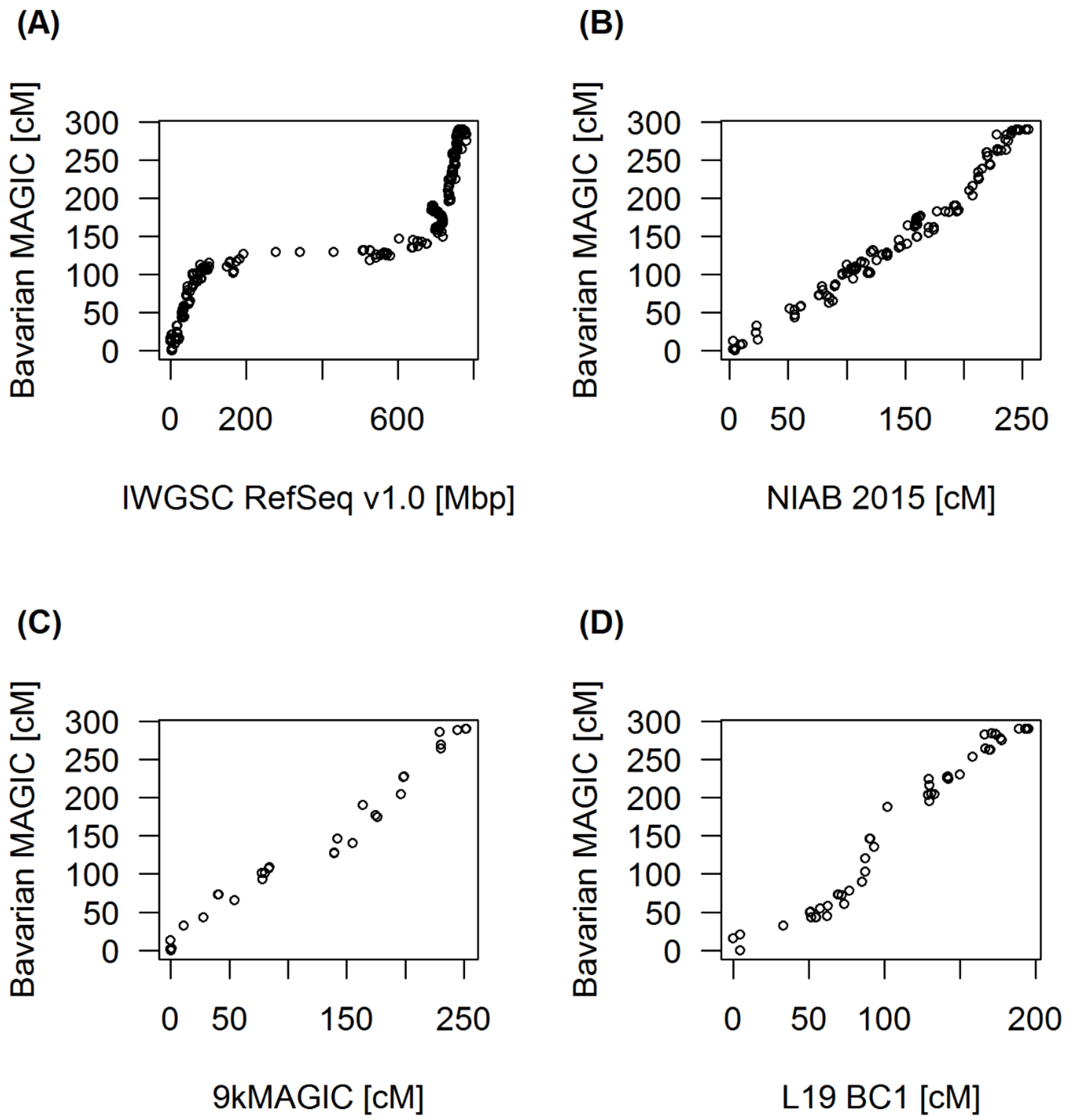

Chromosome 2B

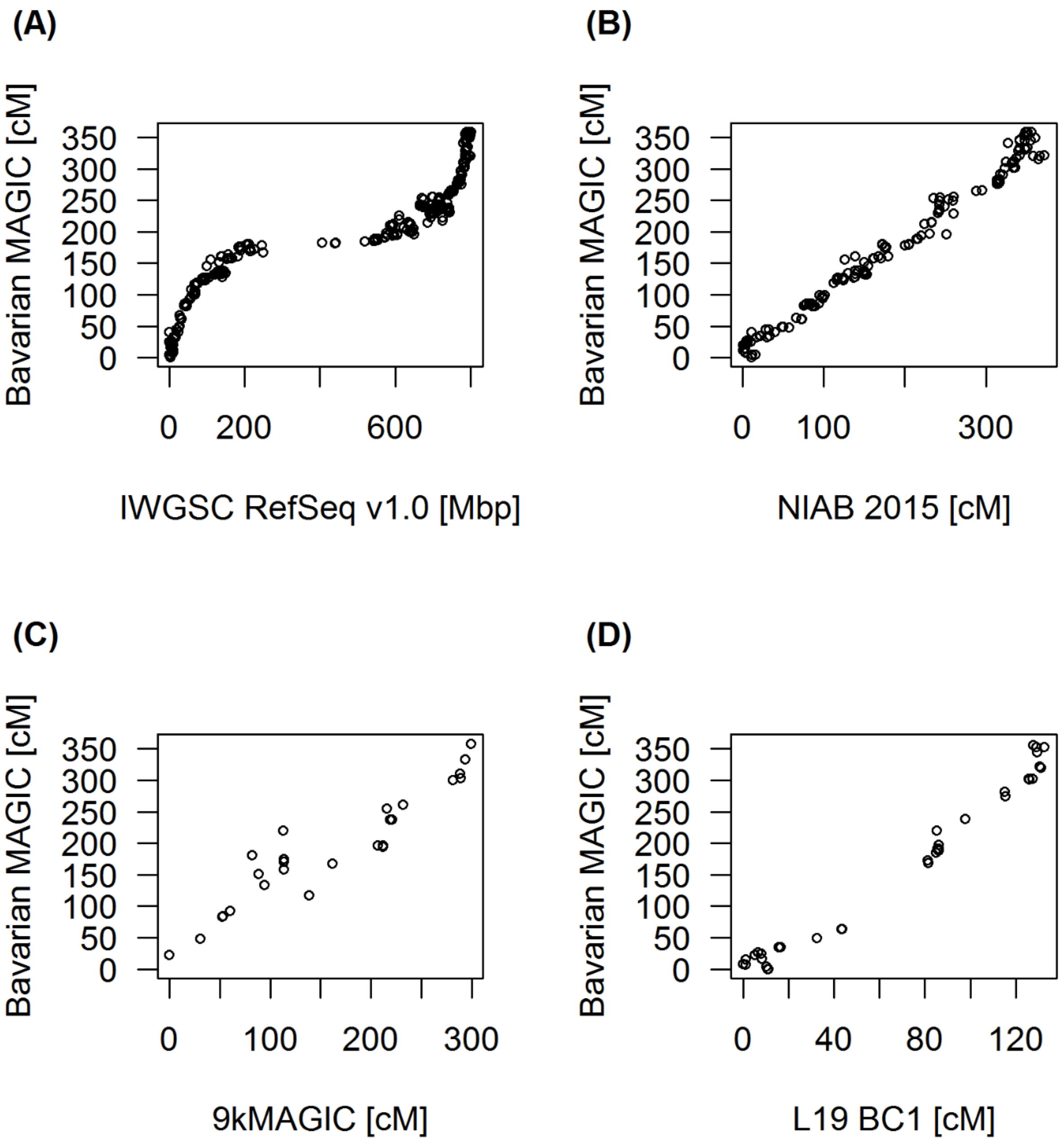

Chromosome 2D

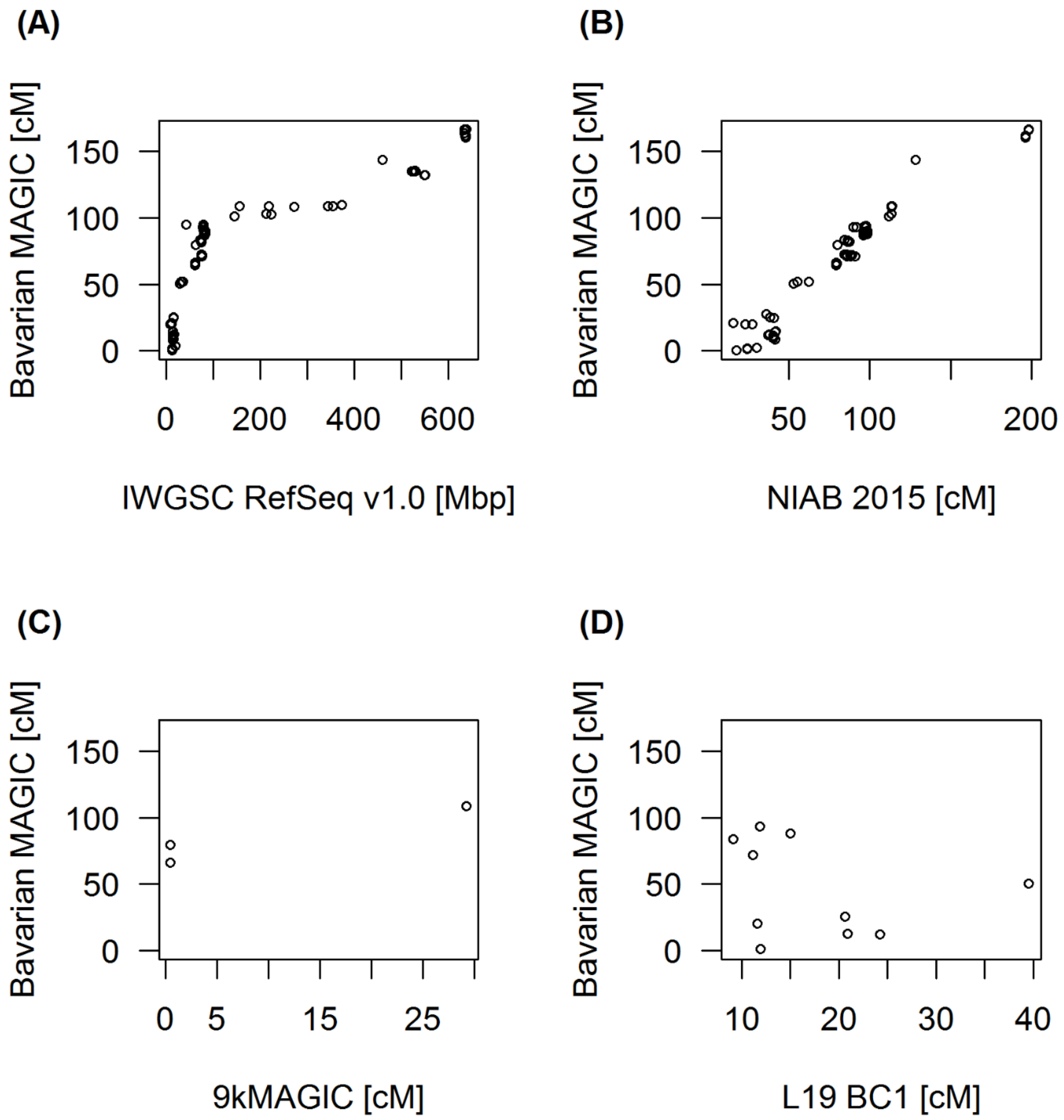

Chromosome 3A

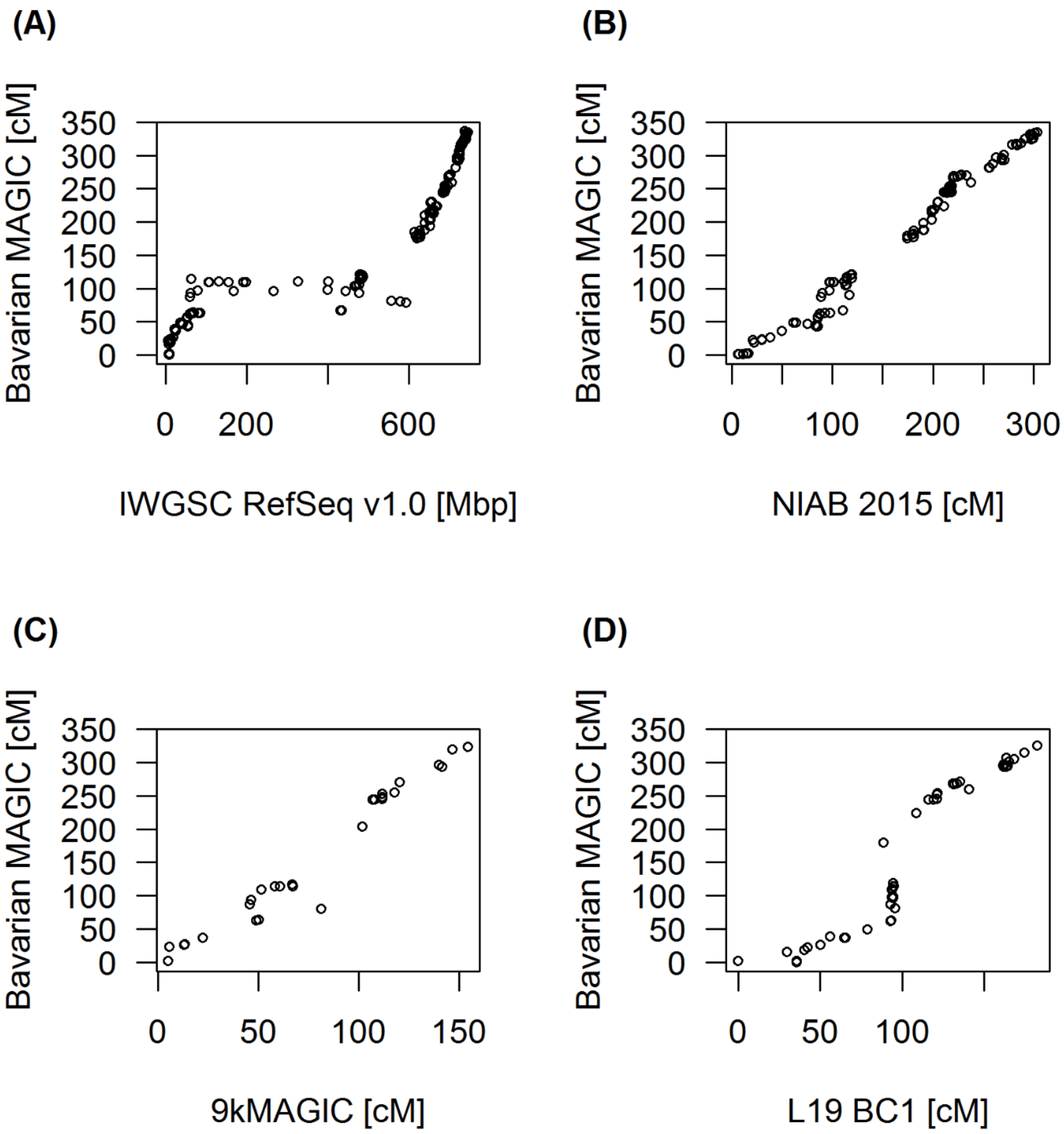

Chromosome 3B

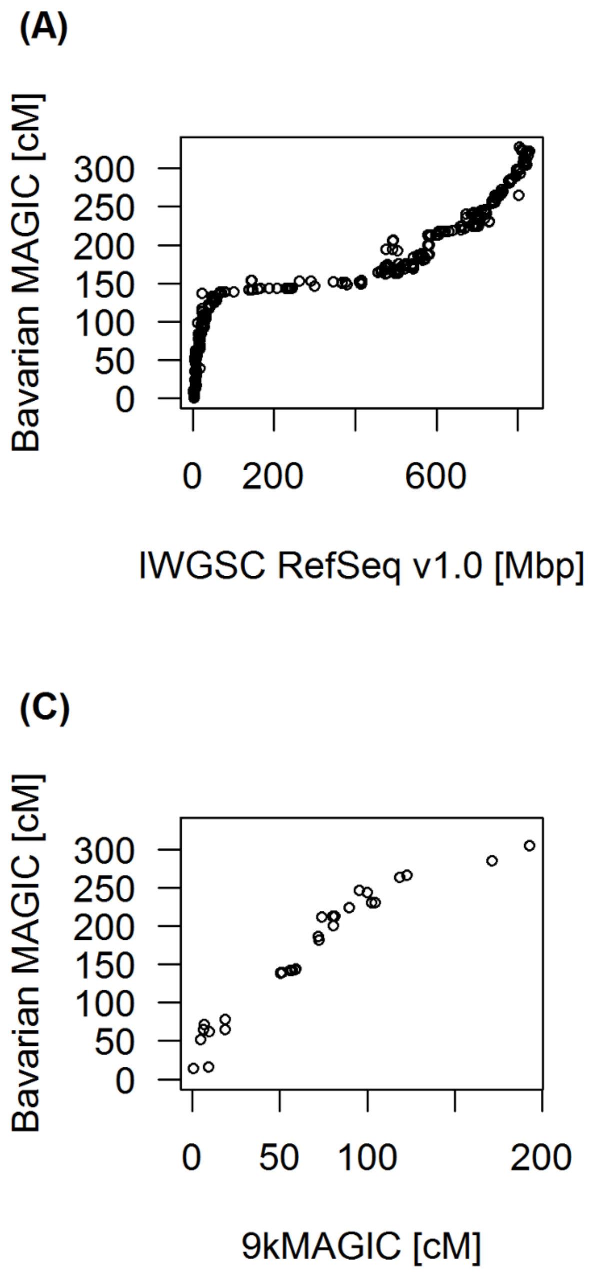

Chromosome 3D

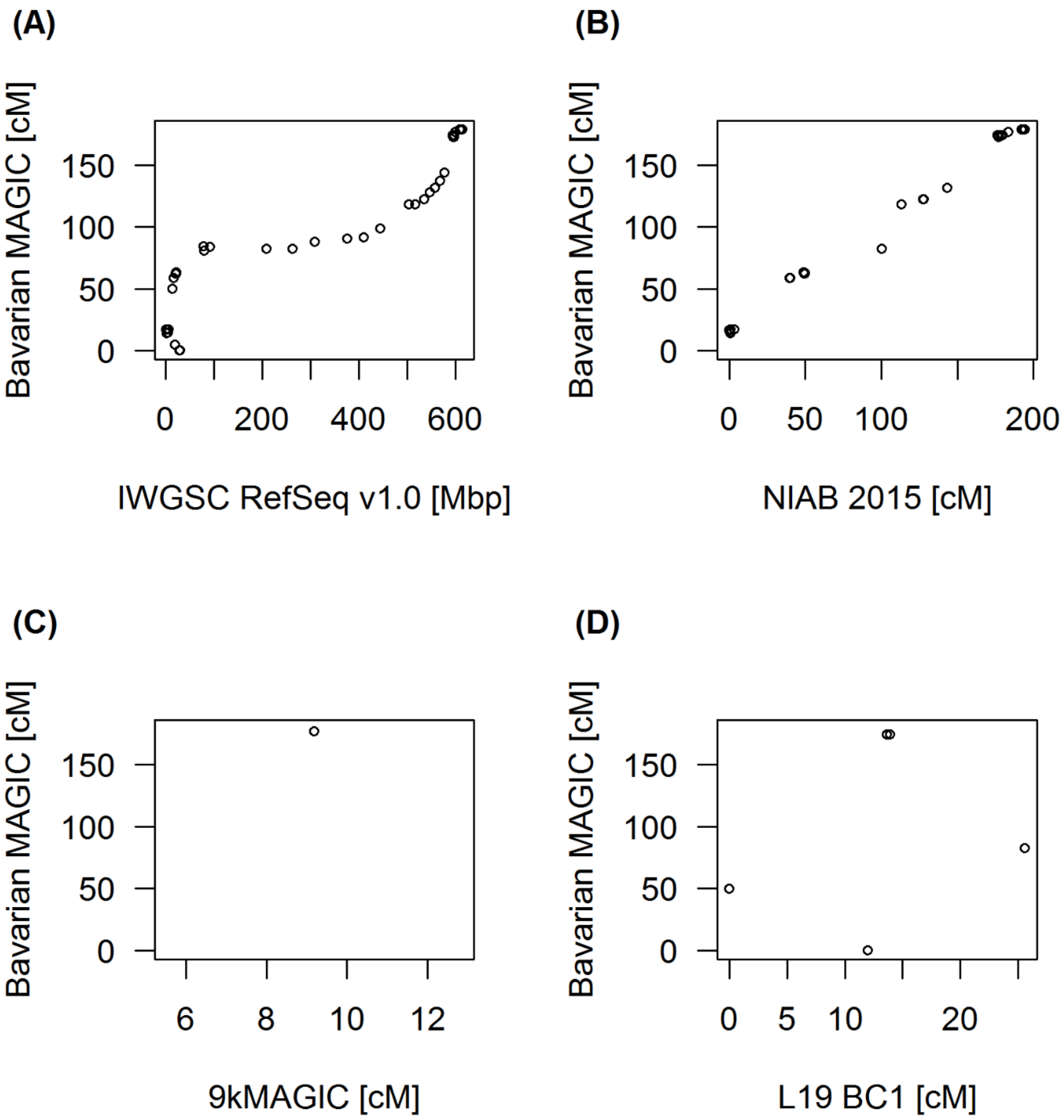

Chromosome 4A

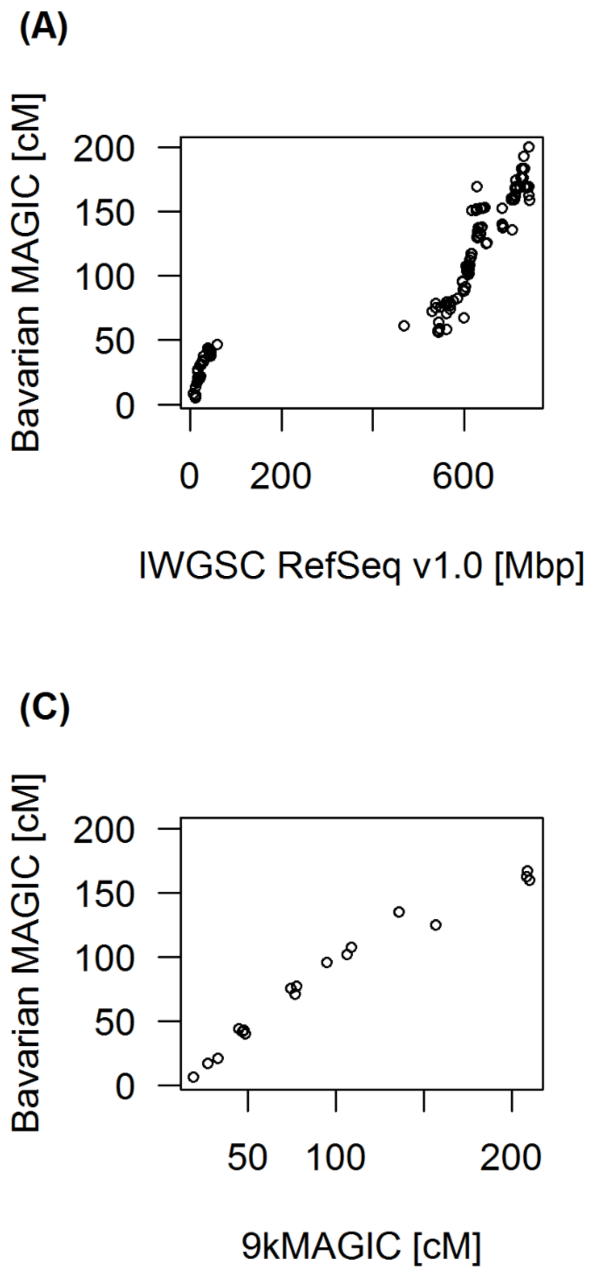

Chromosome 4B

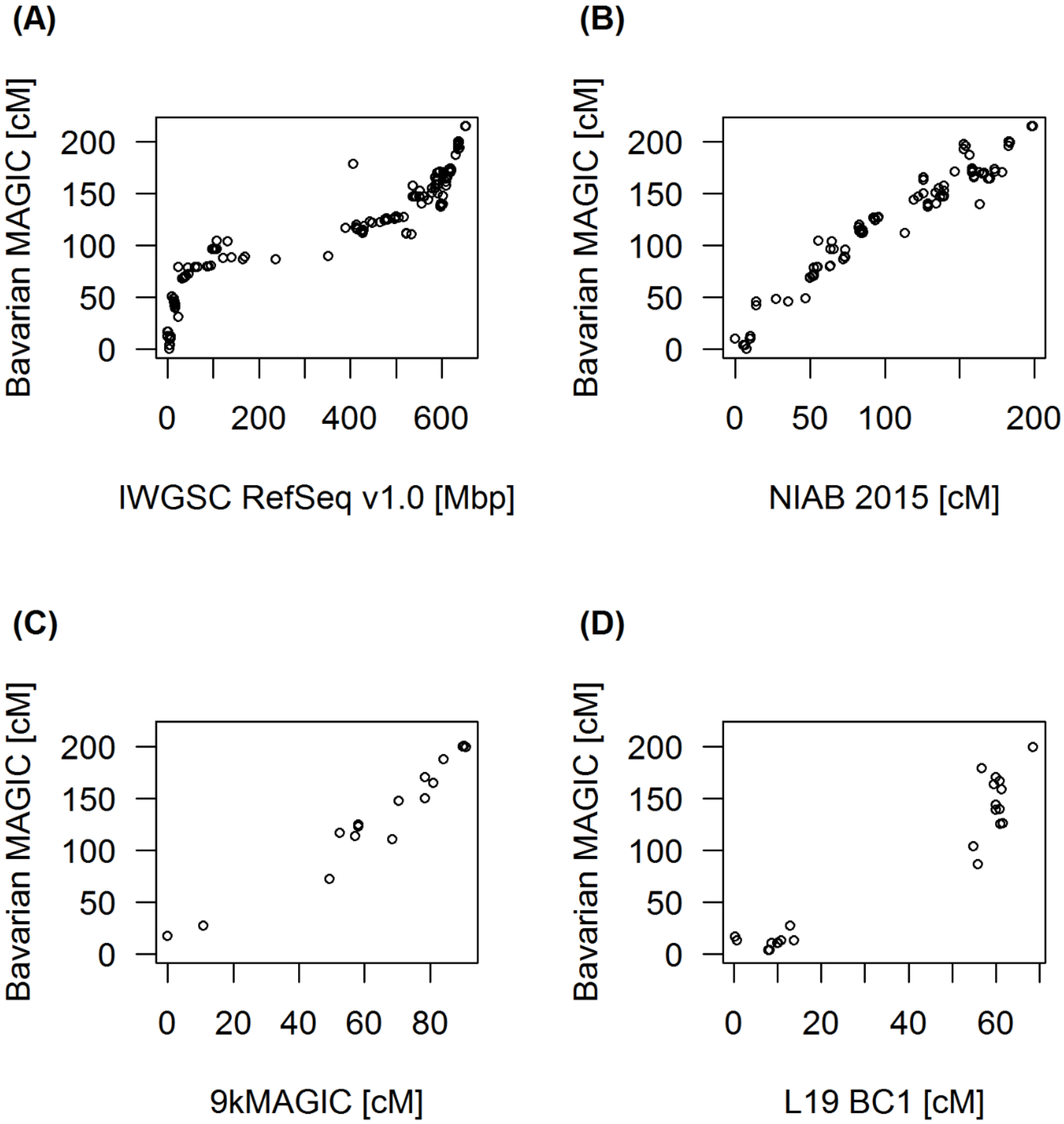

Chromosome 4D

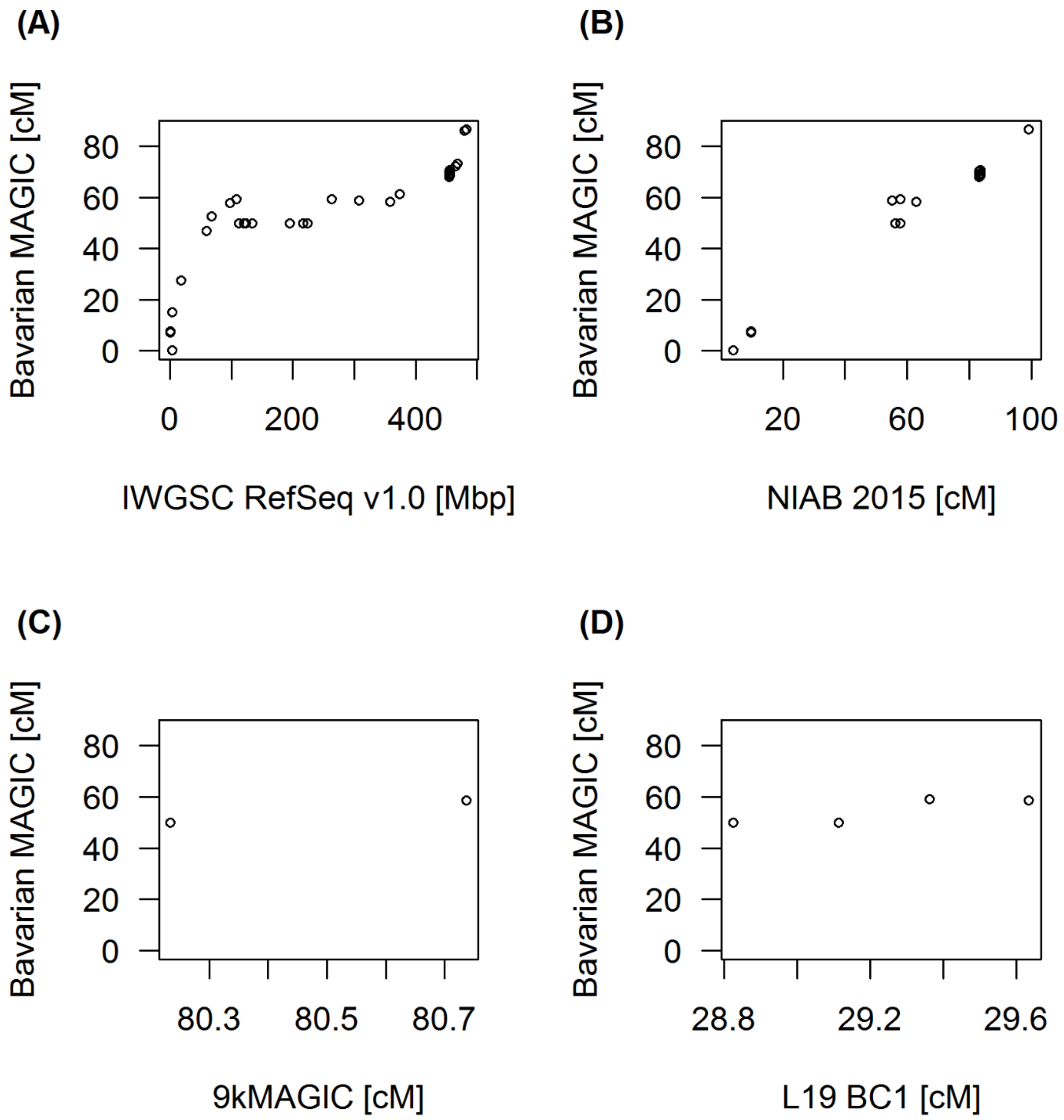

Chromosome 5A

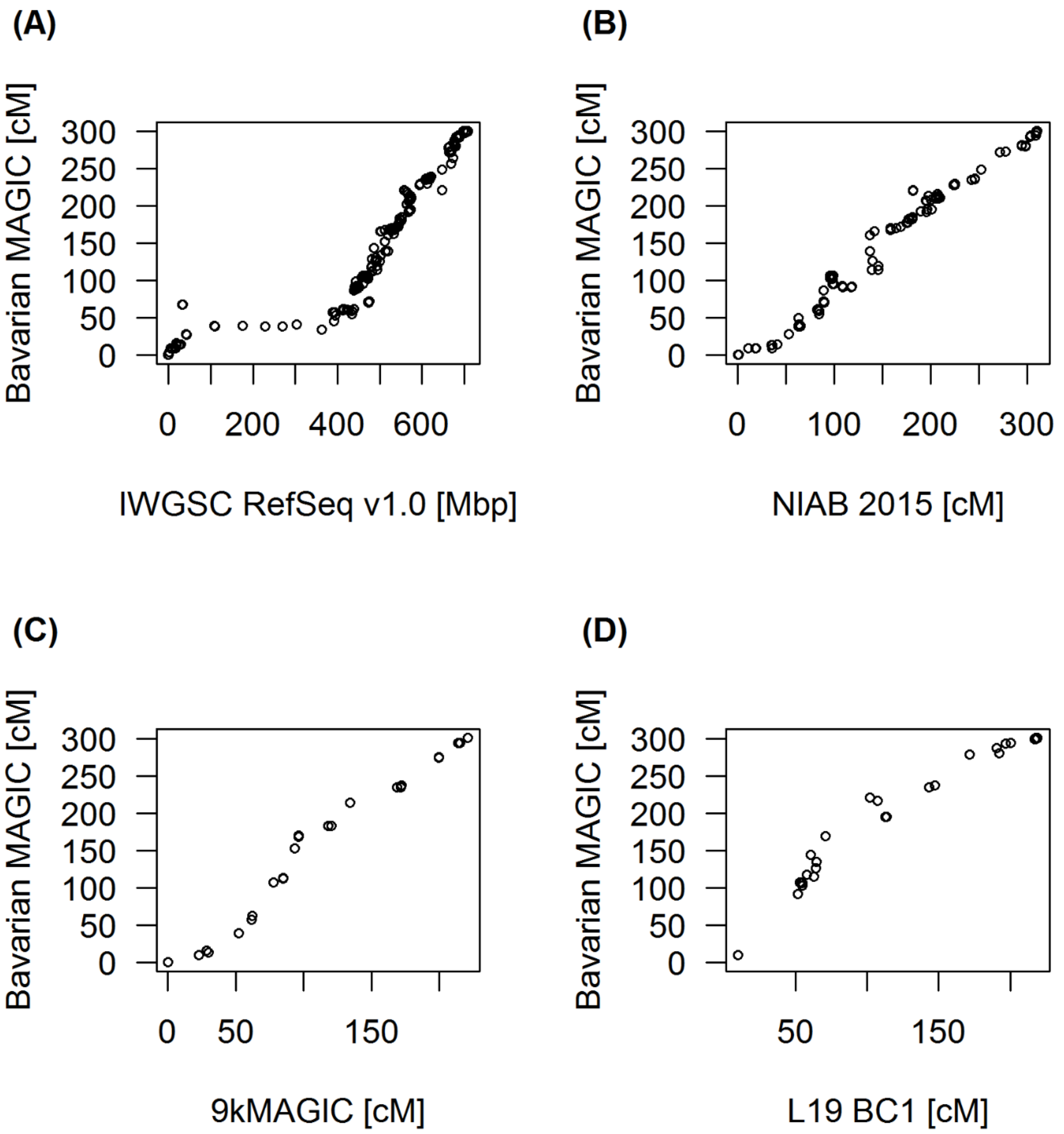

Chromosome 5B

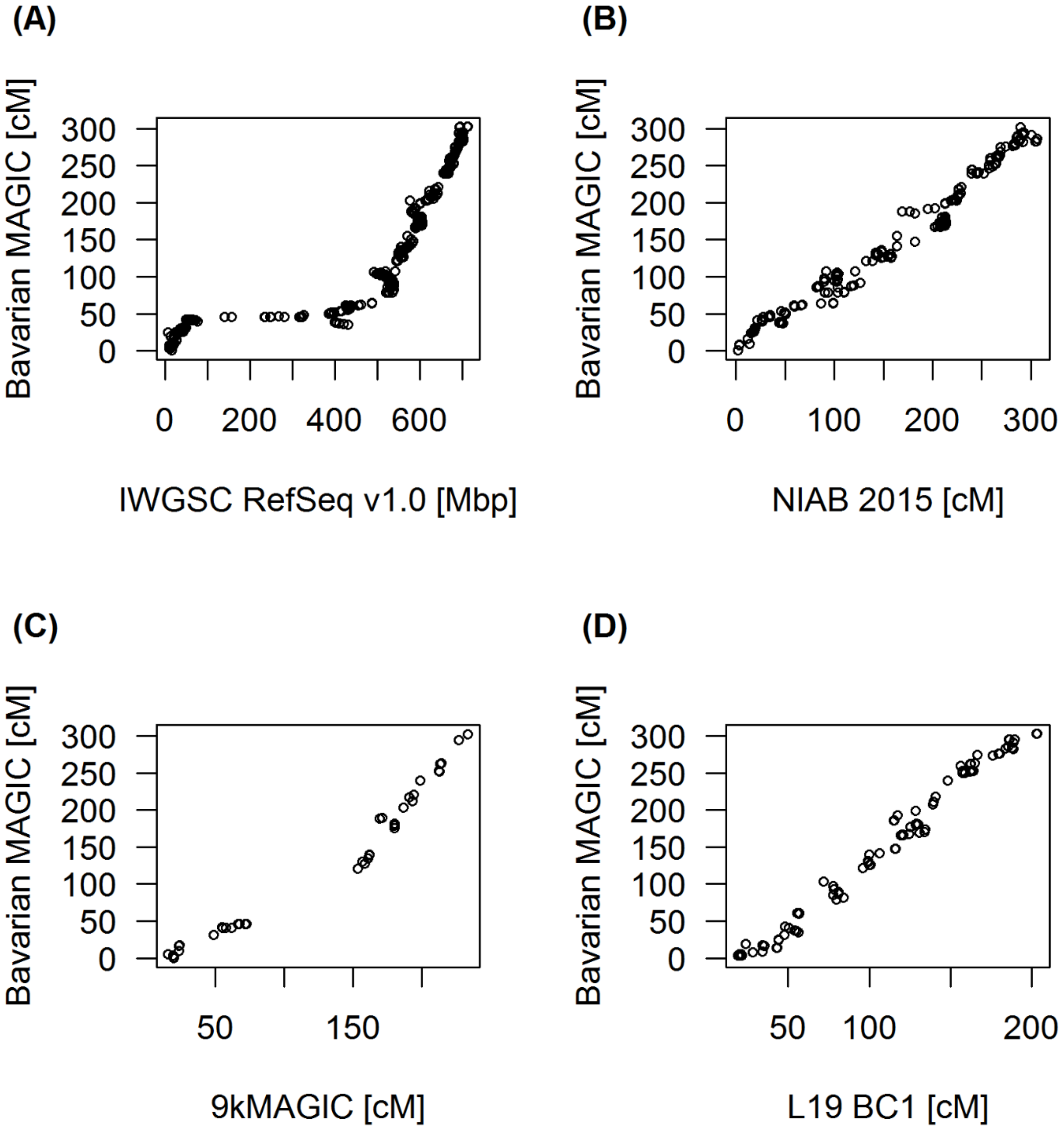

Chromosome 5D

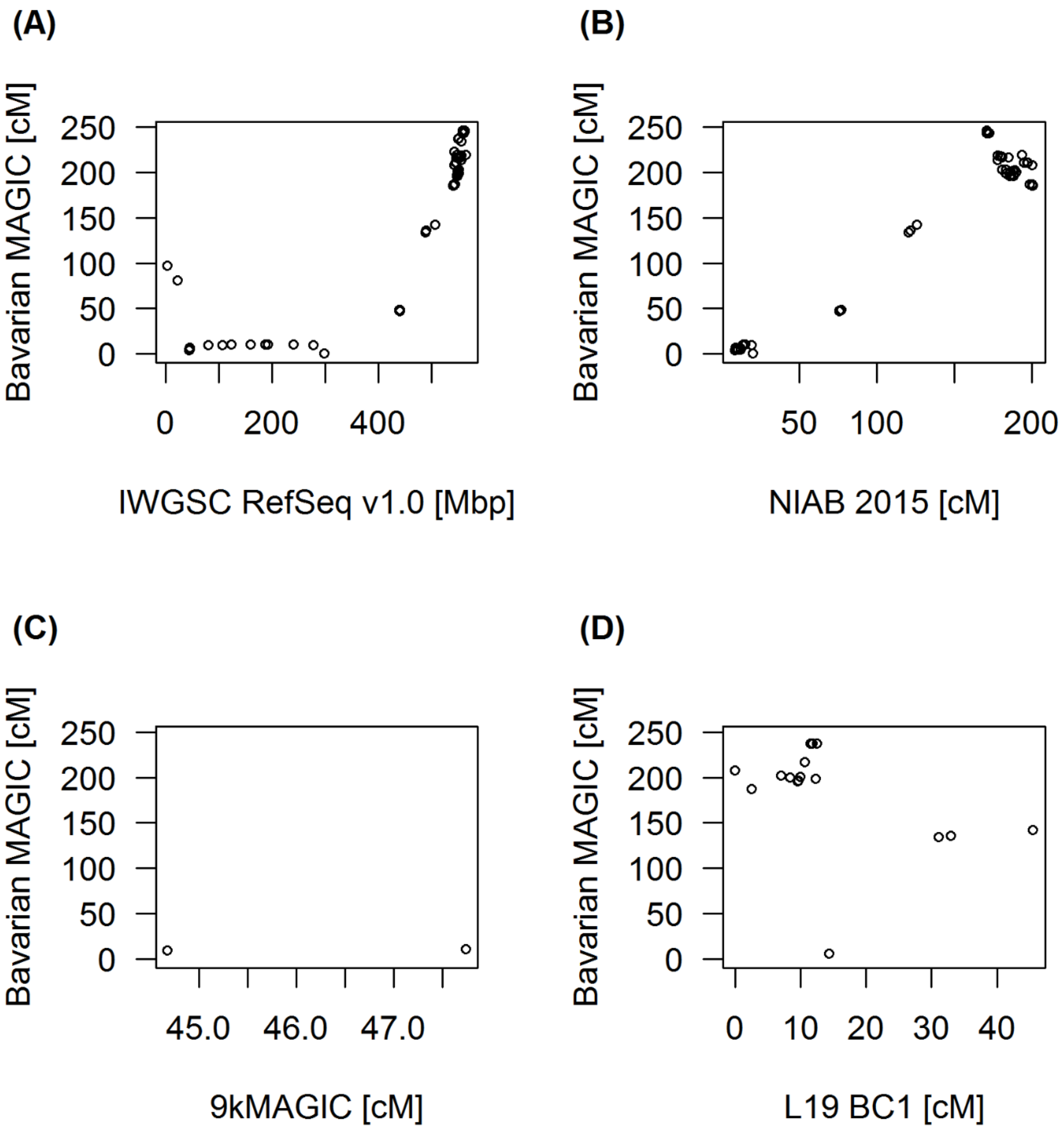

Chromosome 6A

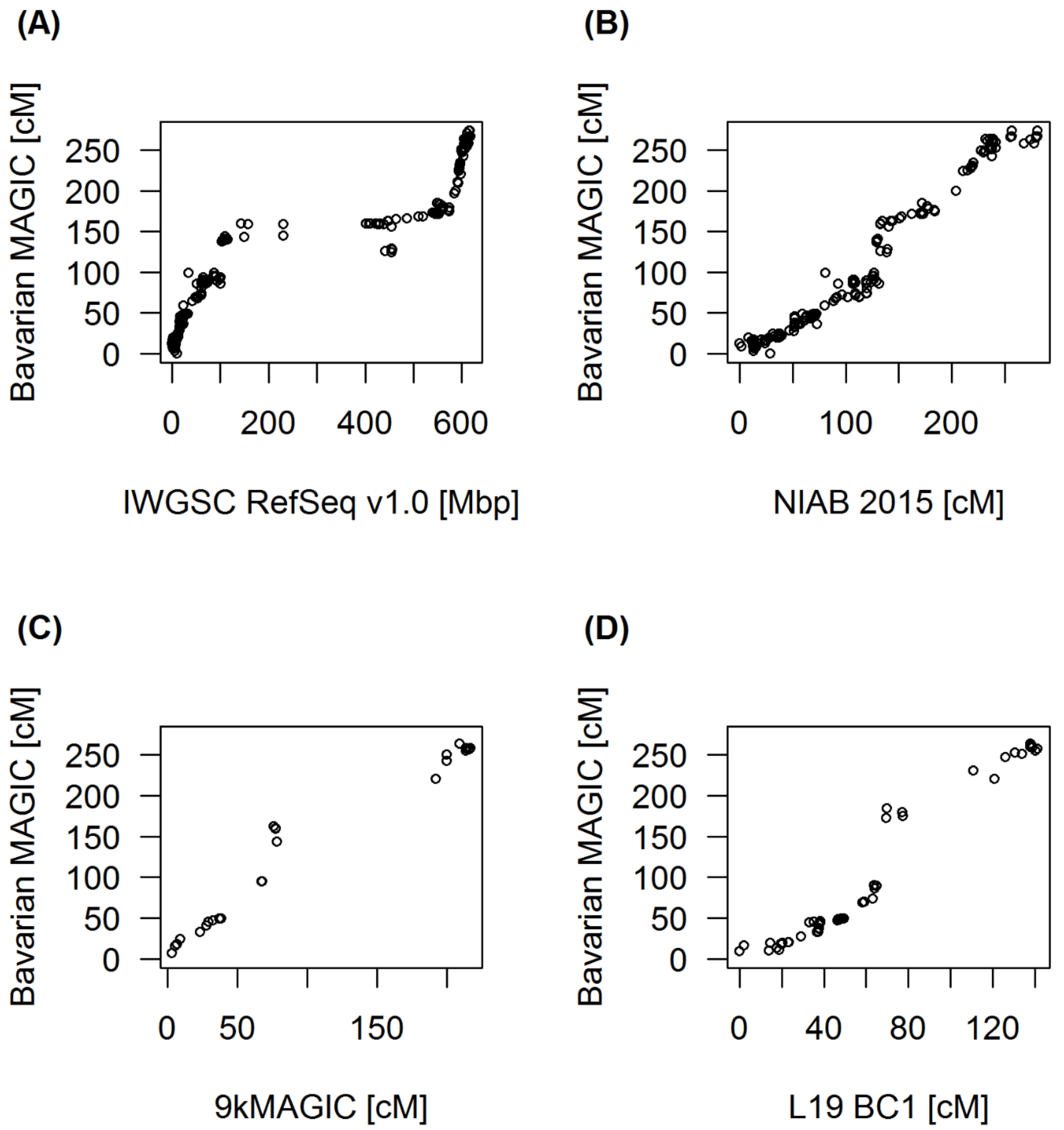

Chromosome 6B

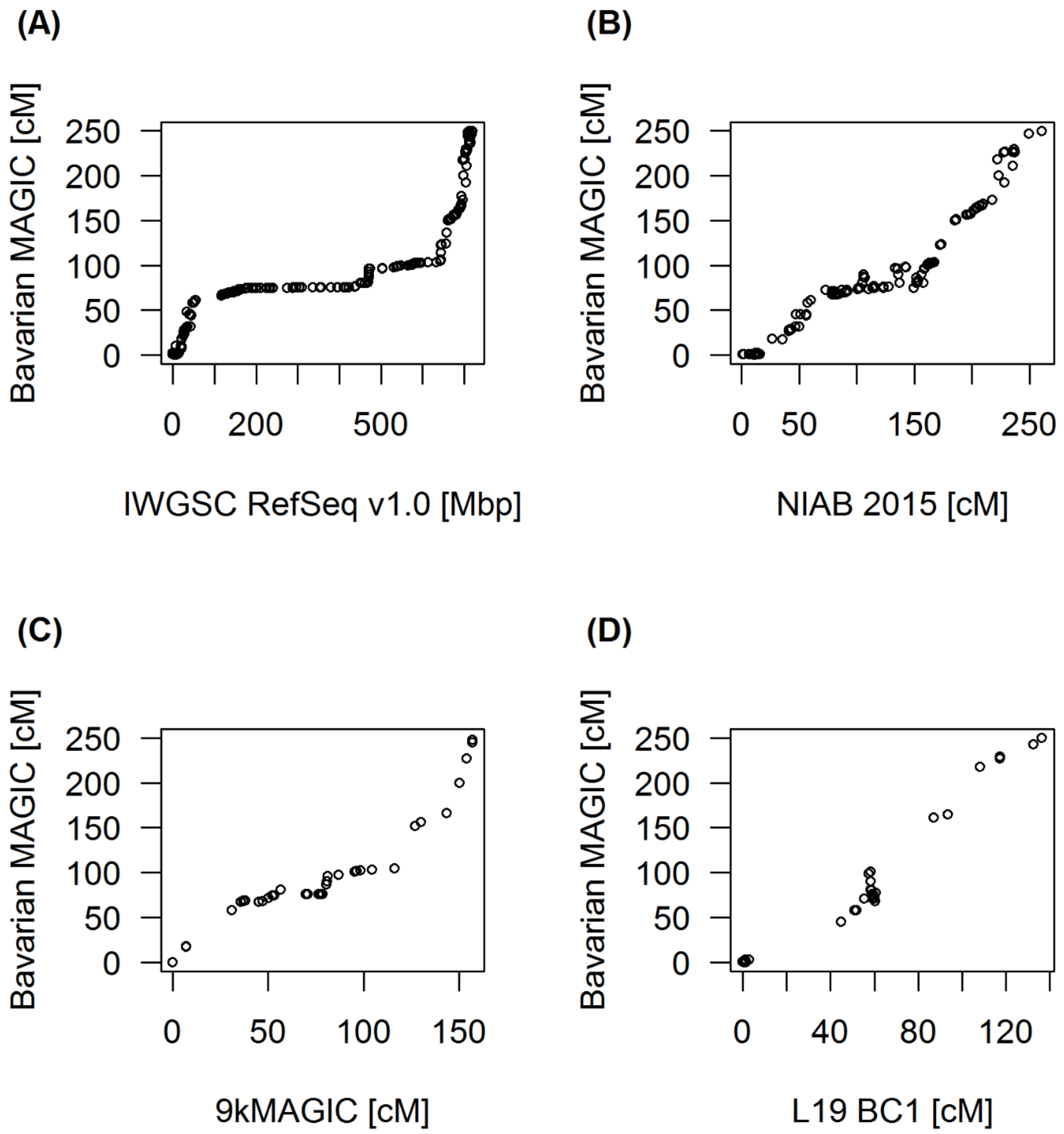

Chromosome 6D

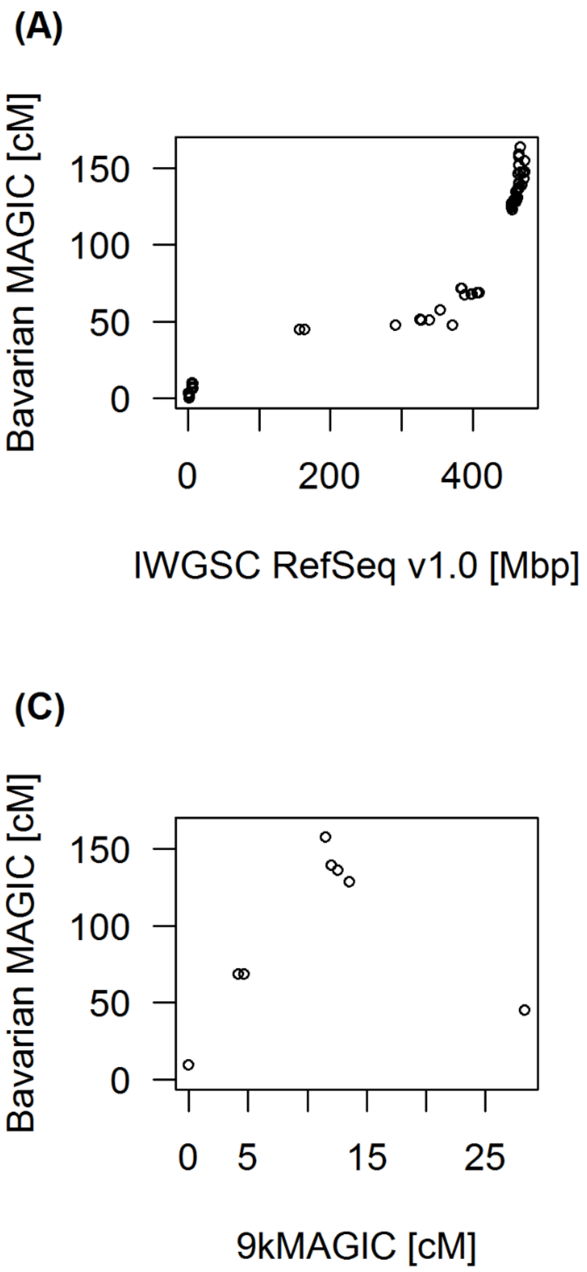

Chromosome 7A

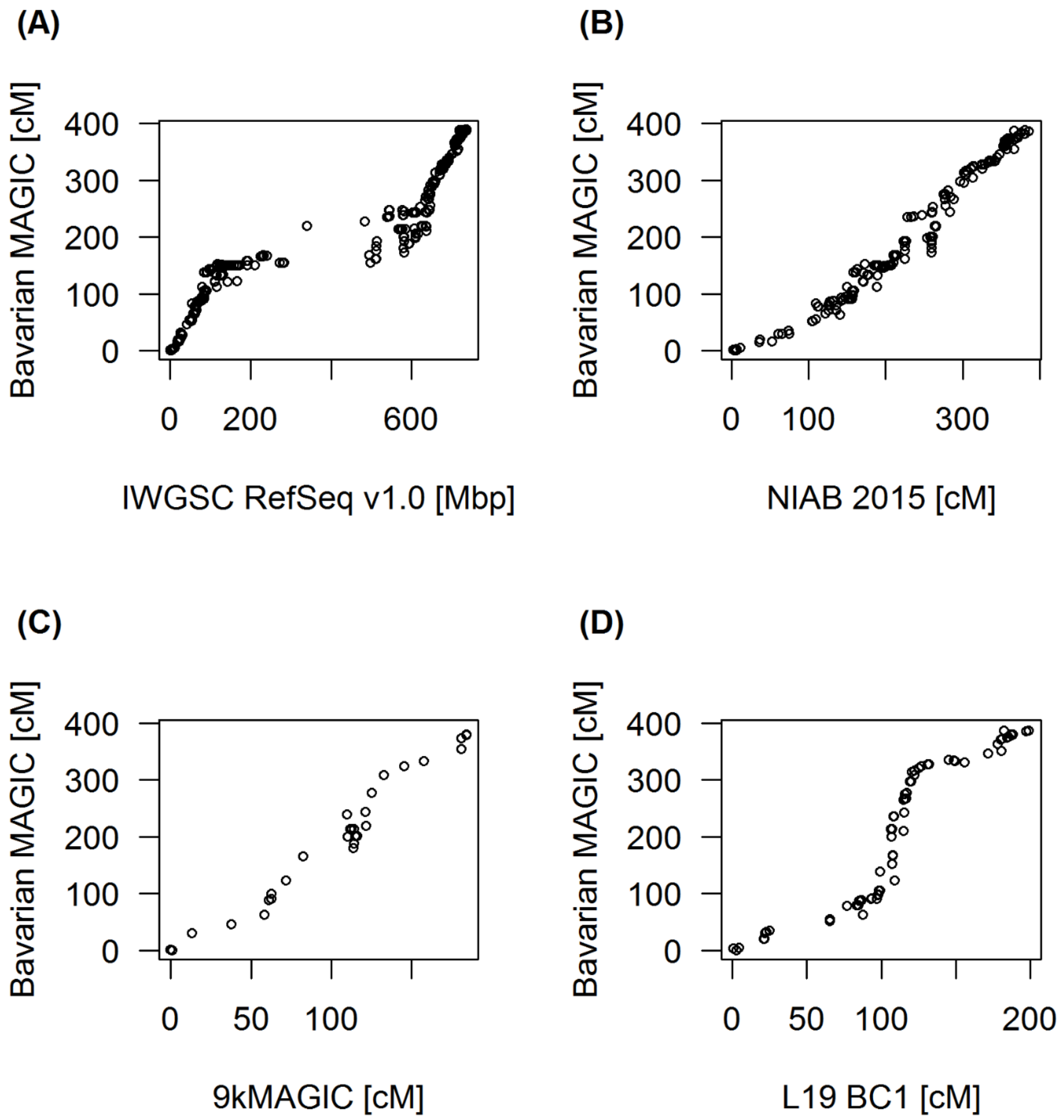

Chromosome 7B

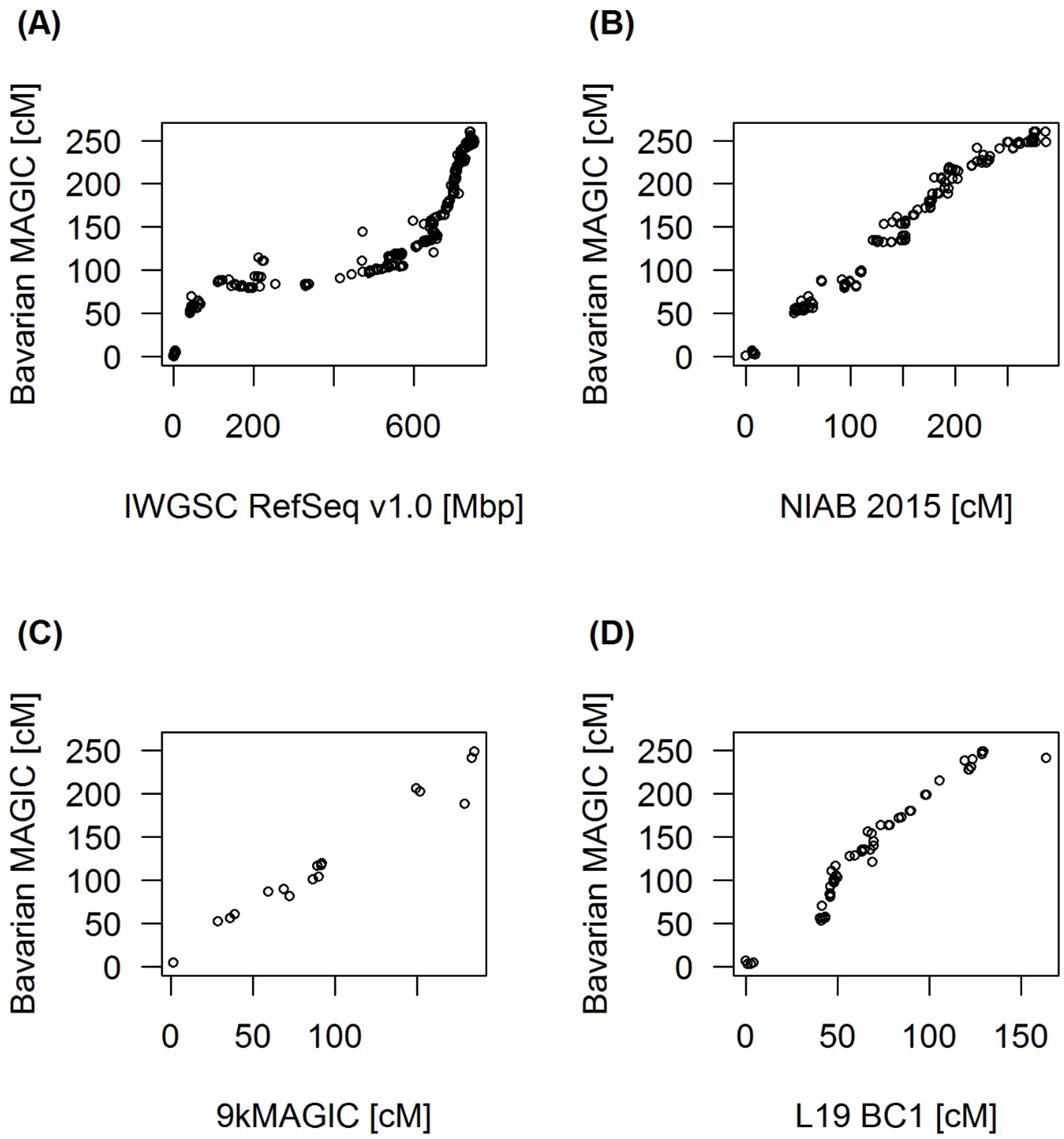

Chromosome 7D

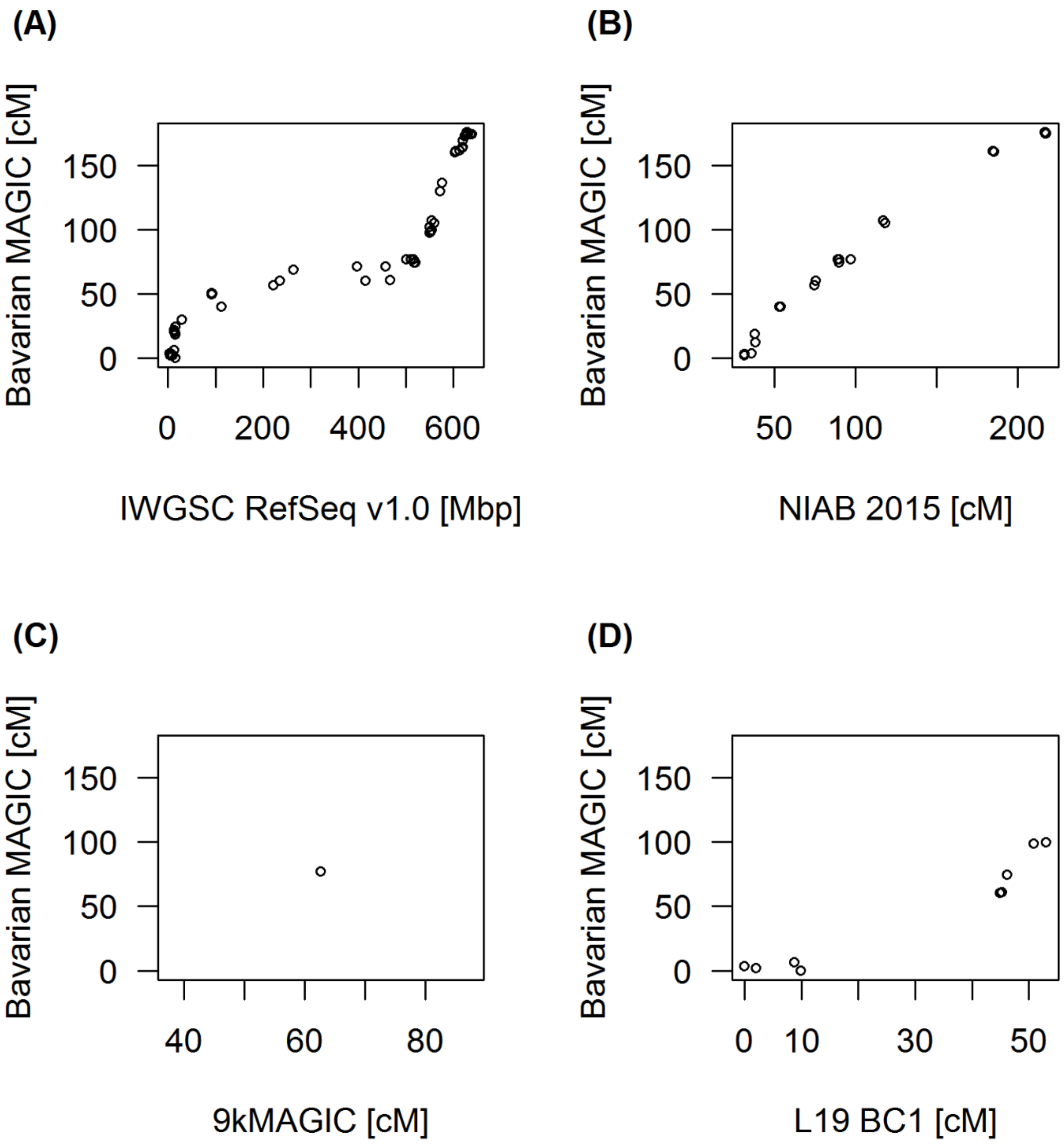

Figure S2: BMWpop marker order comparison. BMWpop marker order compared to (A) physical position of the IWGSC RefSeq v1.0 and the three other genetic maps (B) NIAB 2015, (C) 9kMAGIC, and (D) L19 BC1. BMWpop plotted on y-axis.
