# Supplementary material for: Usefulness of a Multiparent Advanced Generation Intercross Population With a Greatly Reduced Mating Design for Genetic Studies in Winter Wheat
Source: Front Plant Sci. 2018 Dec 6;9:1825. doi: 10.3389/fpls.2018.01825 (PMC6291512; doi:10.3389/fpls.2018.01825)
Supplement: Supplementary file 3 [file Data_Sheet_3.PDF]

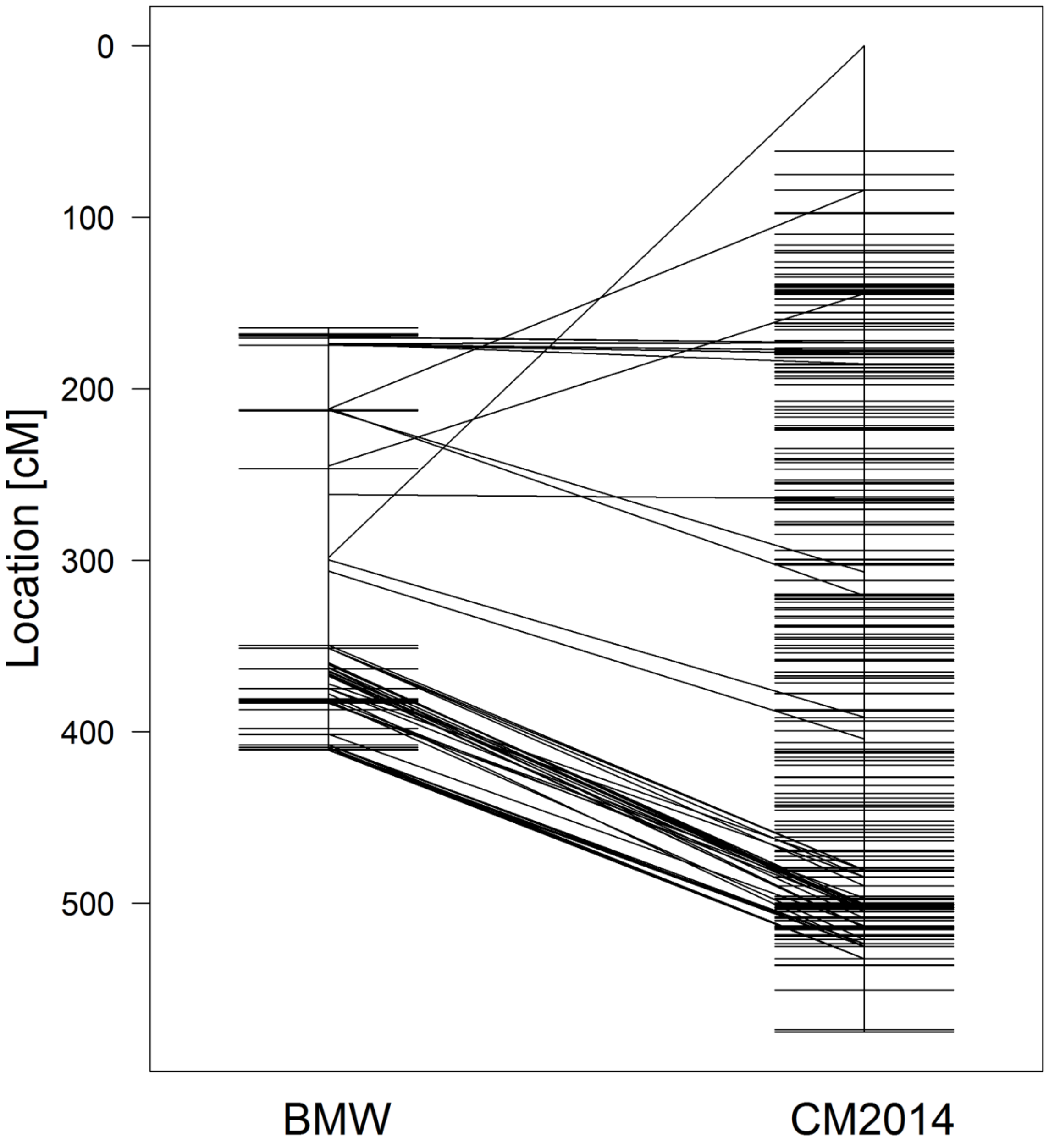

Figure S3: Comparison of chromosome 5D between BMWpop and CM2014 consensus linkage map (Wang et al. 2014). Short horizontal lines indicate unique markers per map, remaining lines connect congruent markers on both maps.
