# Supplementary material for: Usefulness of a Multiparent Advanced Generation Intercross Population With a Greatly Reduced Mating Design for Genetic Studies in Winter Wheat
Source: Front Plant Sci. 2018 Dec 6;9:1825. doi: 10.3389/fpls.2018.01825 (PMC6291512; doi:10.3389/fpls.2018.01825)
Supplement: Supplementary file 4 [file Data_Sheet_4.PDF]

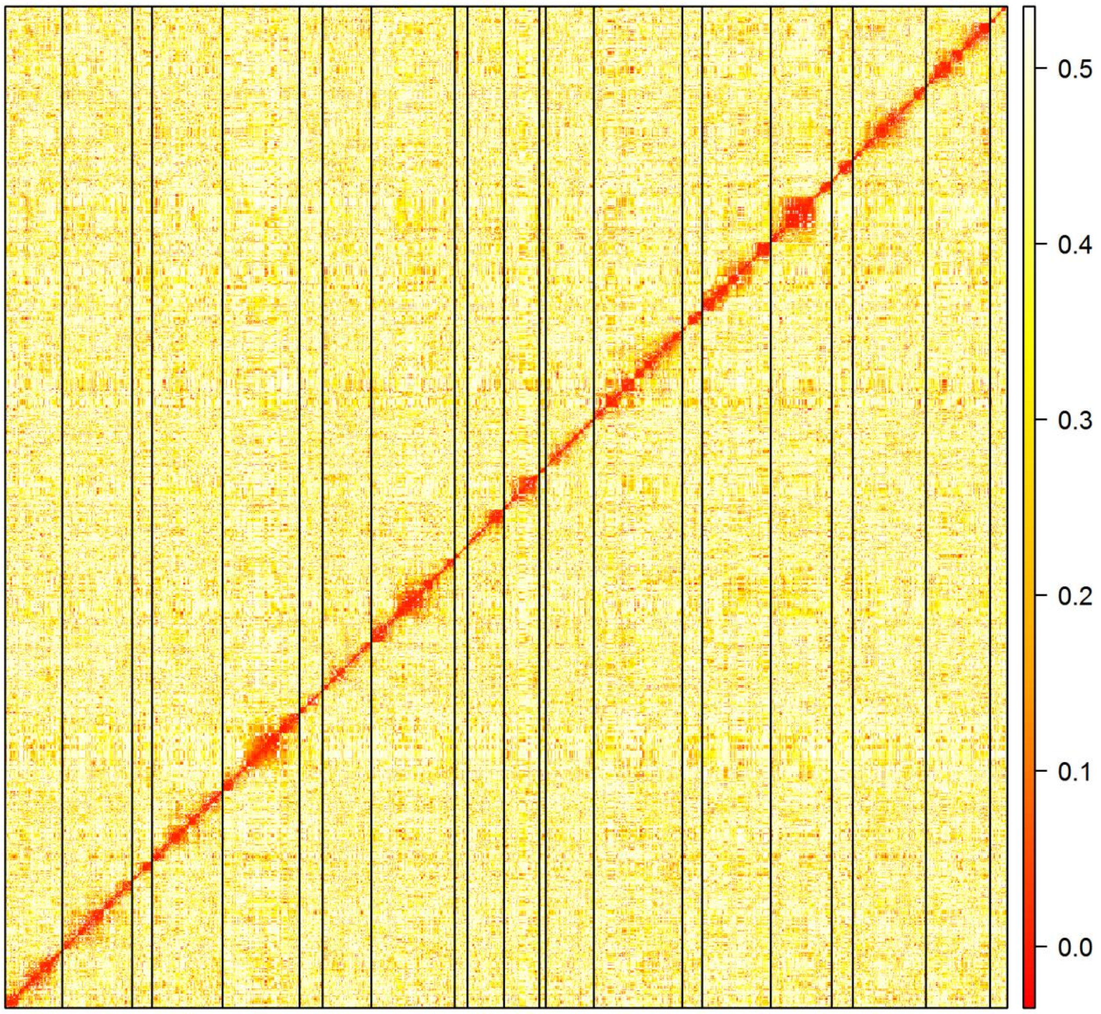

Figure S4: BMWpop recombination fraction diagnostics of whole genome. Red color indicates low (0) and white color high recombination rate (0.5). Overall heatmap of the recombination fraction matrix starting bottom left with chromosome 1A, 1B, and ending up top right with chromosome 7D. Vertical lines represent the border between two chromosomes.
