# Supplementary material for: Usefulness of a Multiparent Advanced Generation Intercross Population With a Greatly Reduced Mating Design for Genetic Studies in Winter Wheat
Source: Front Plant Sci. 2018 Dec 6;9:1825. doi: 10.3389/fpls.2018.01825 (PMC6291512; doi:10.3389/fpls.2018.01825)
Supplement: Supplementary file 5 [file Data_Sheet_5.PDF]

## Chromosome 1

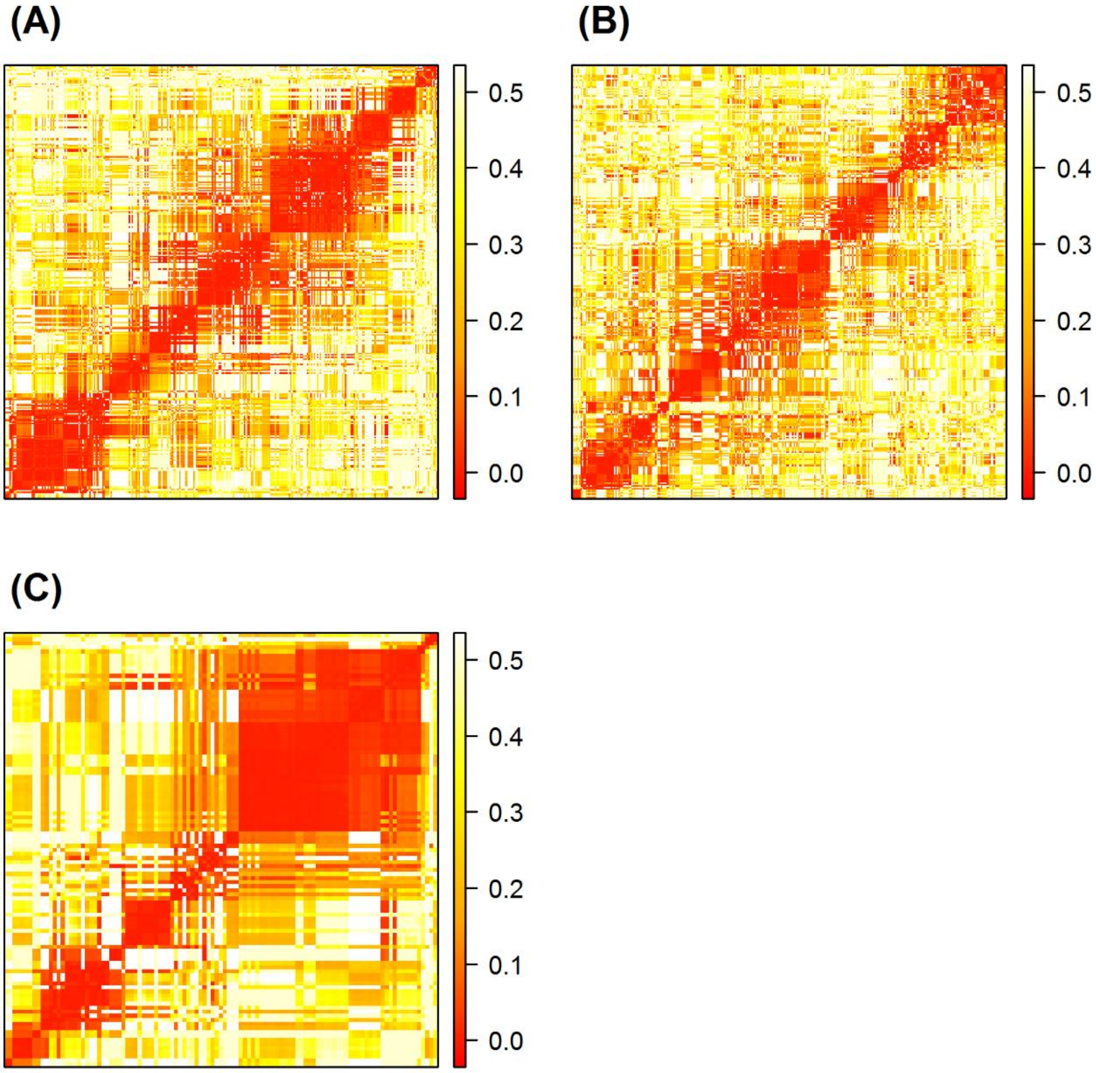

## Chromosome 2

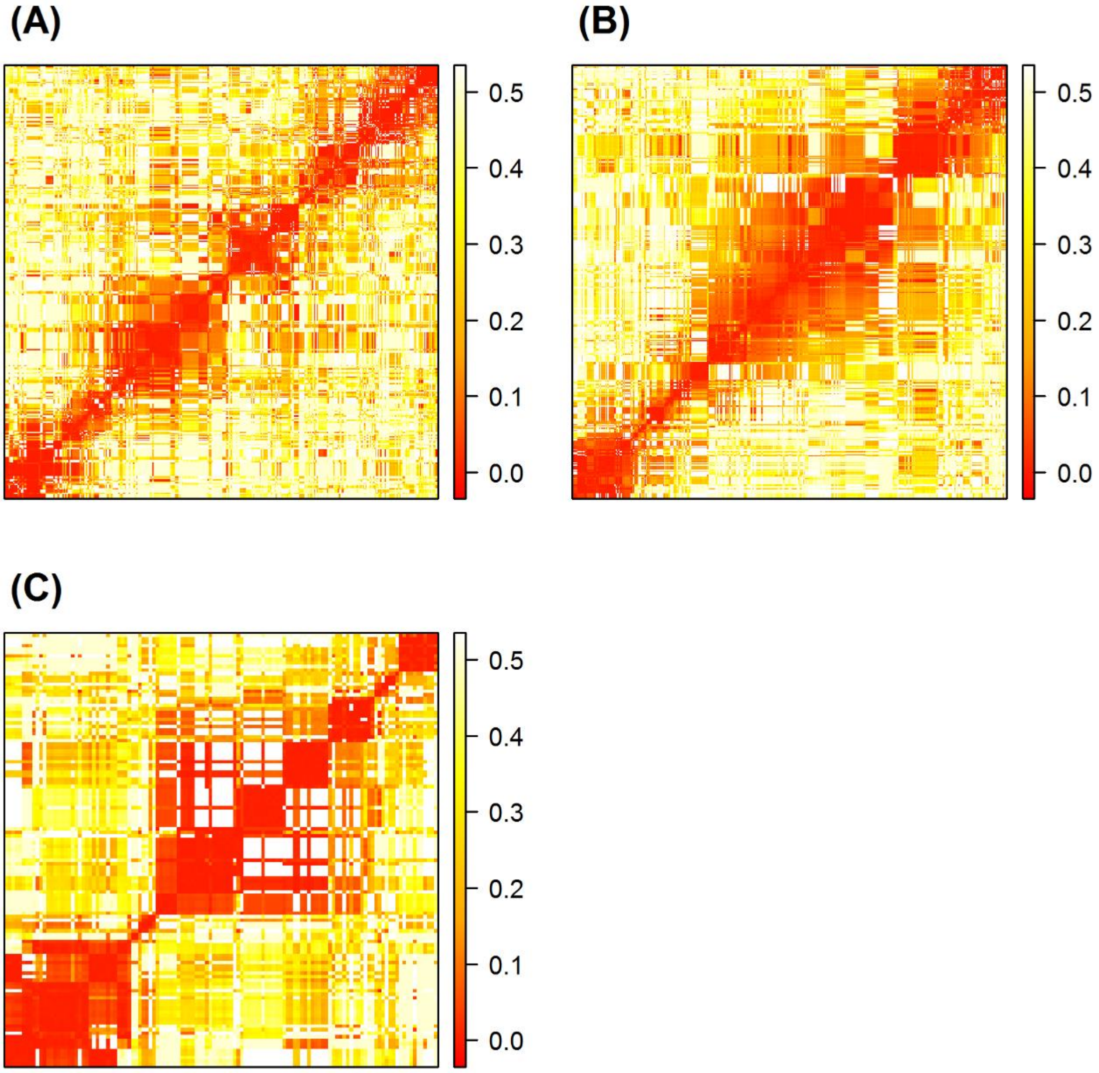

**Chromosome 3**

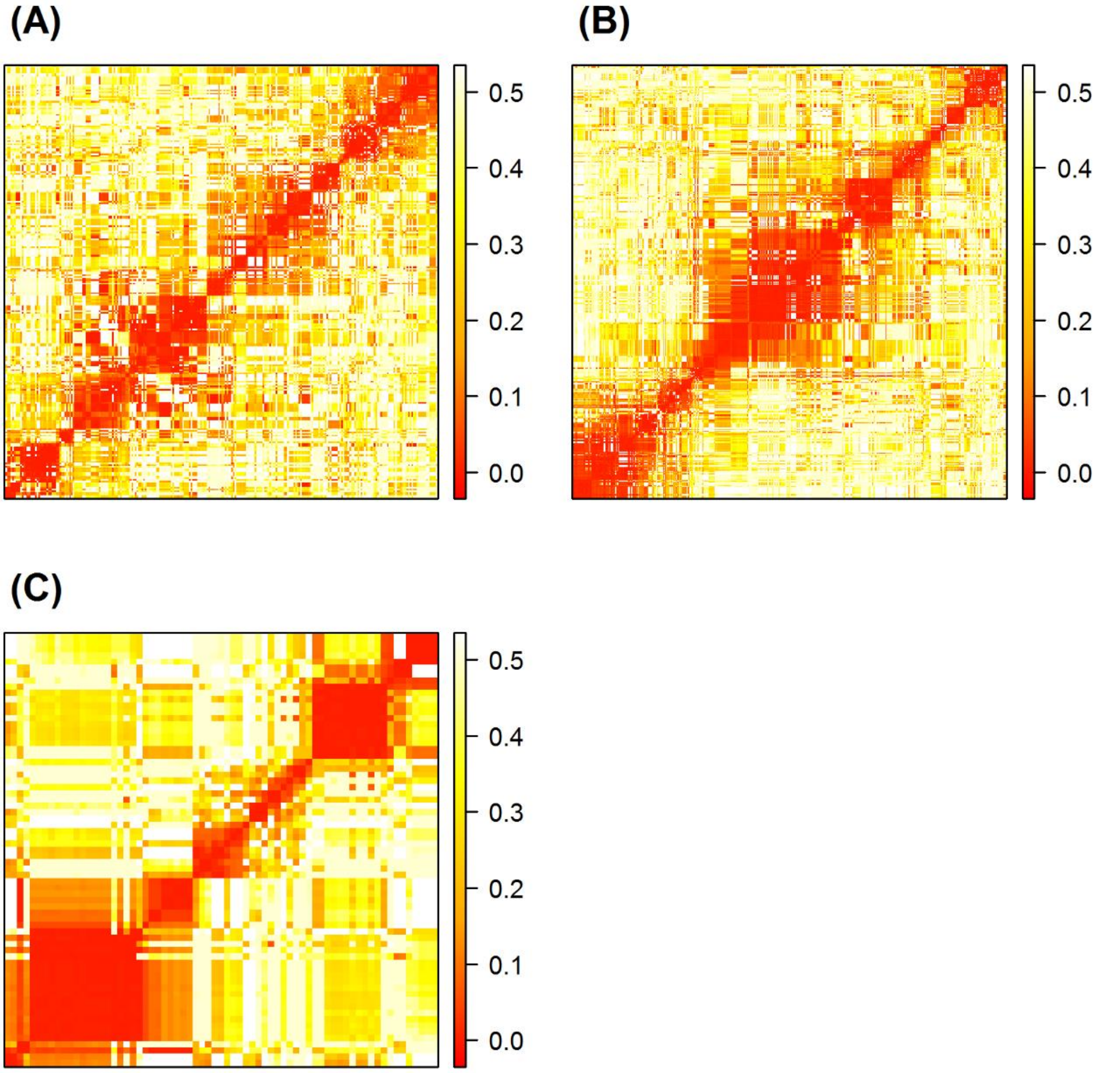

## Chromosome 4

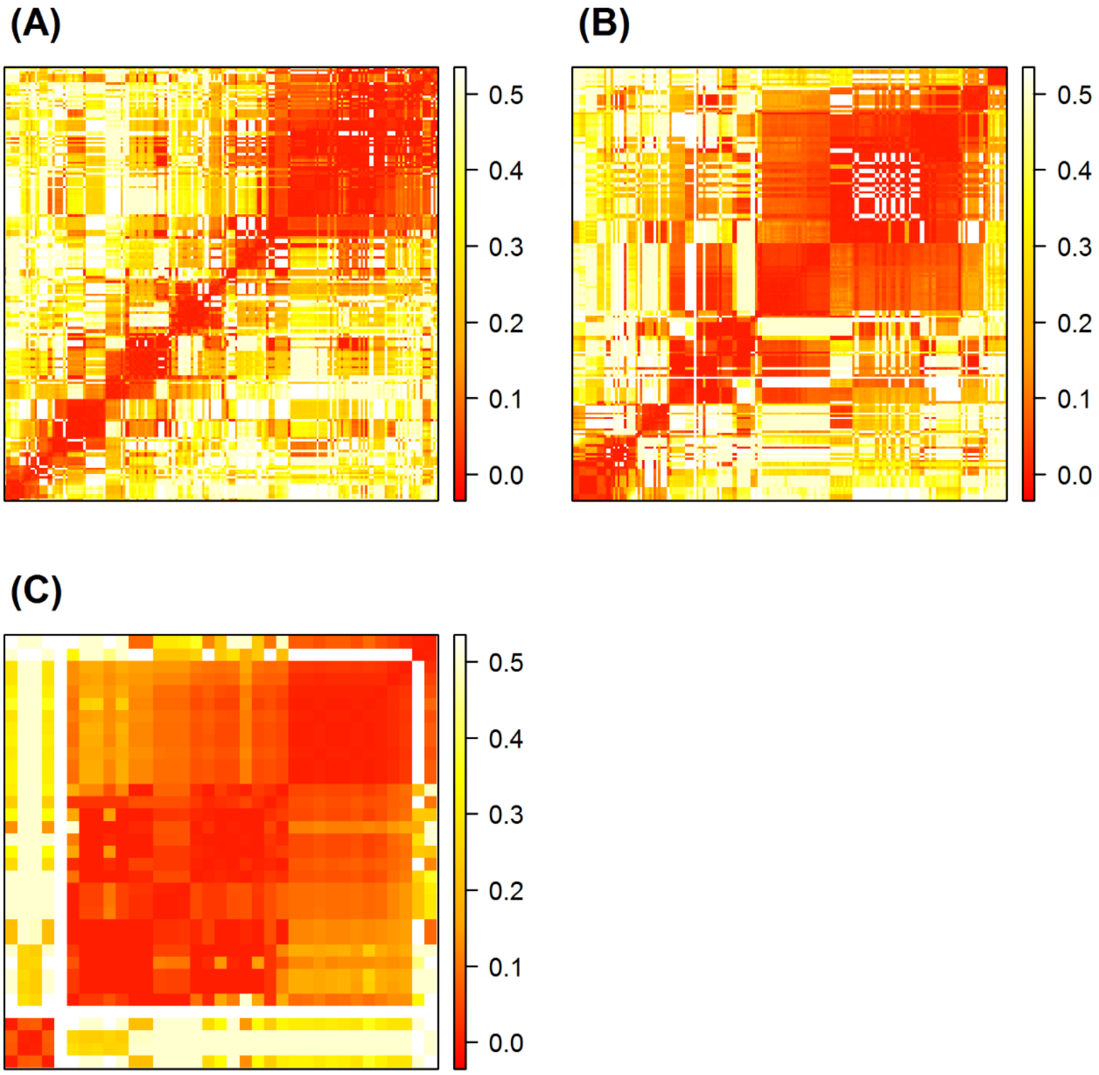

## Chromosome 5

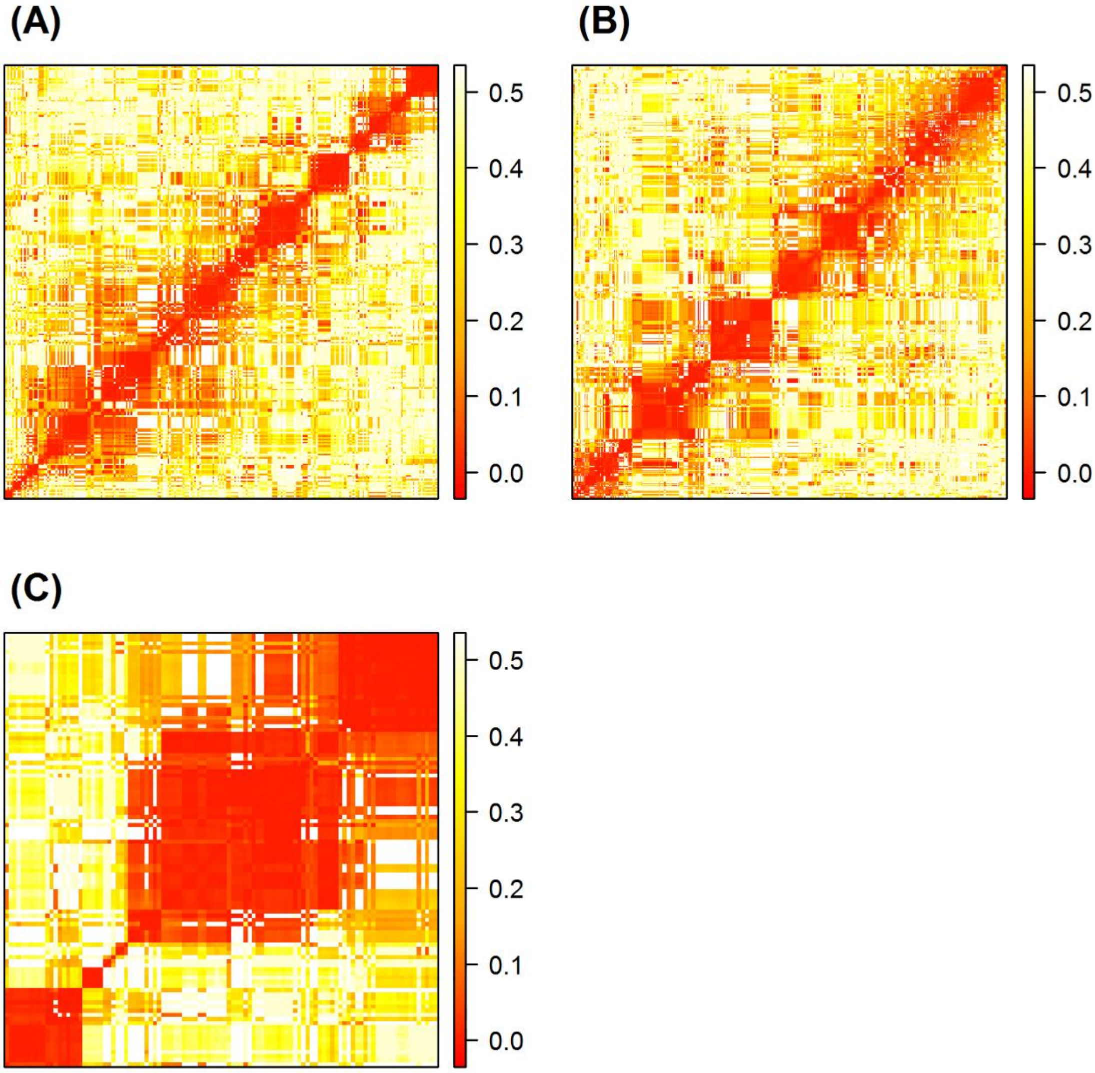

**Chromosome 6**

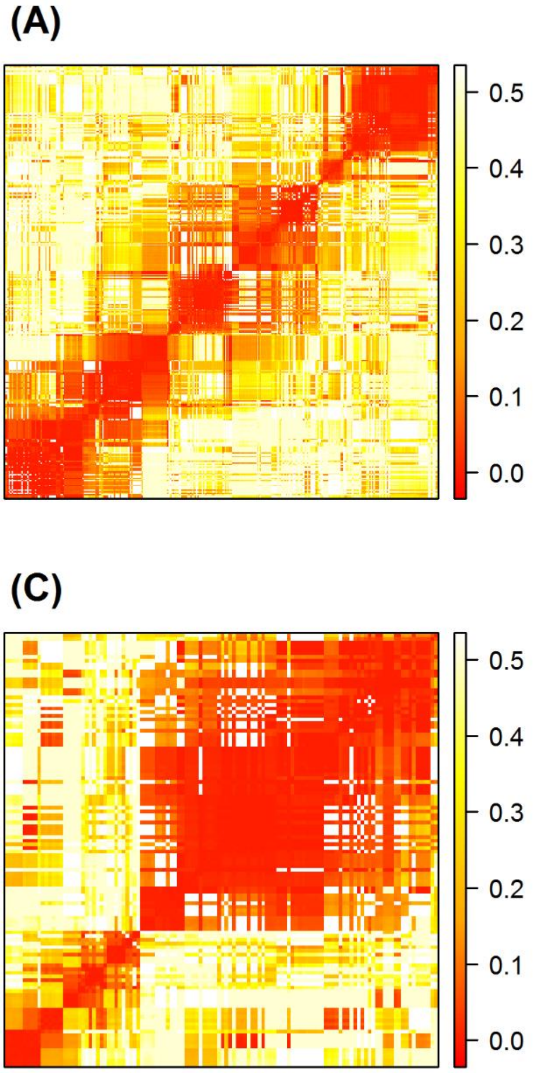

**Chromosome 7**

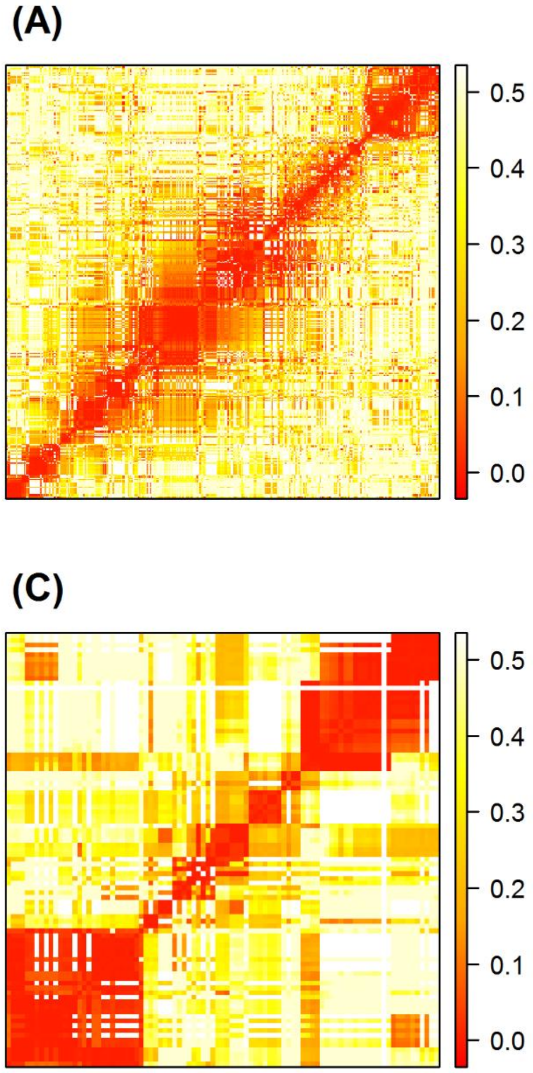


**Figure S5:** Recombination fraction diagnostics. Red color indicates low (0) and white color high recombination rate (0.5). (A), (B), and (C) show individual heatmaps of chromosomes for A, B, and D genome, respectively.
