# Supplementary material for: Usefulness of a Multiparent Advanced Generation Intercross Population With a Greatly Reduced Mating Design for Genetic Studies in Winter Wheat
Source: Front Plant Sci. 2018 Dec 6;9:1825. doi: 10.3389/fpls.2018.01825 (PMC6291512; doi:10.3389/fpls.2018.01825)
Supplement: Supplementary file 6 [file Data_Sheet_6.PDF]

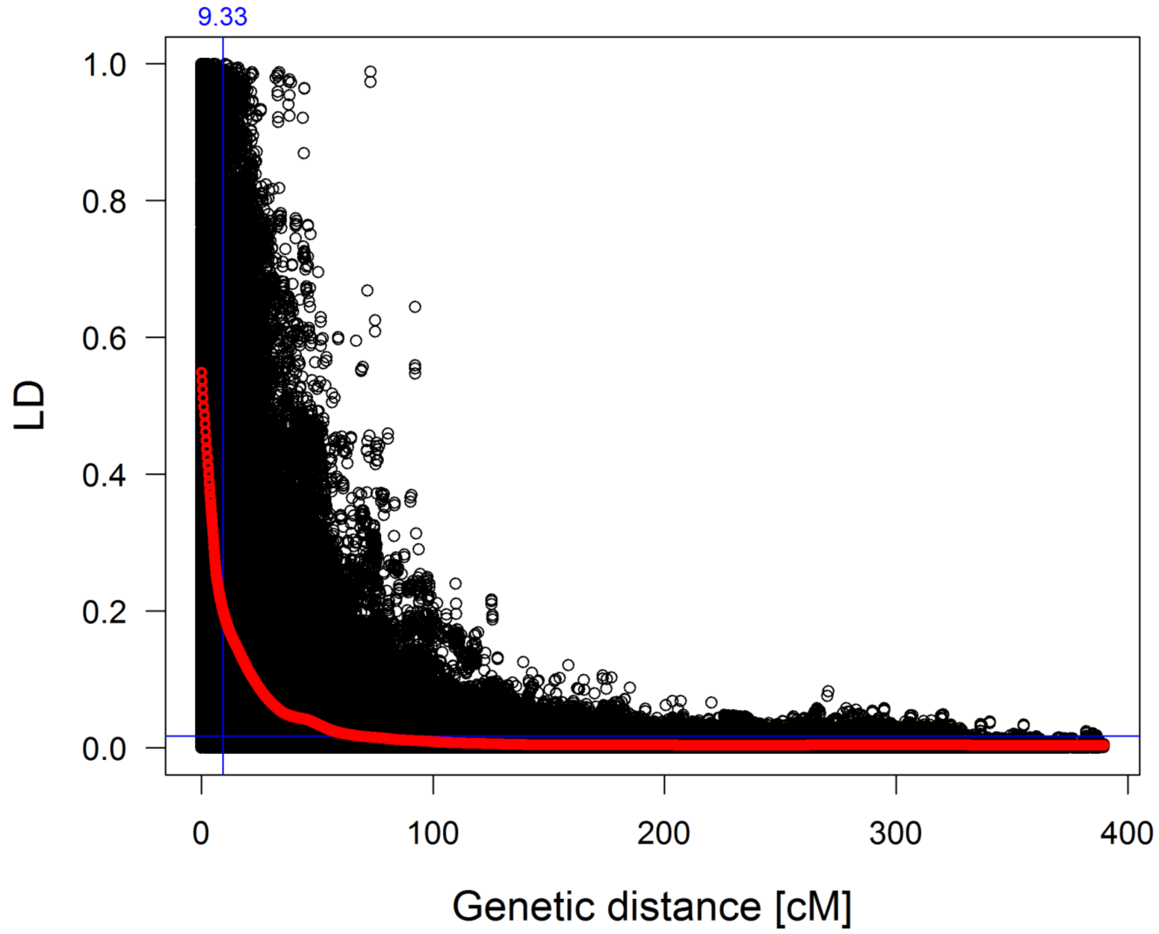

Figure S6: Intra-chromosomal linkage disequilibrium (LD) decay for the genome (all chromosomes). Horizontal blue line indicates the population-specific critical r^2 value (0.017). Vertical blue line indicates genetic distance where LD decreased to $r^2 < 0.2$. Red line marks the fitted loess curve.
