# Supplementary material for: Usefulness of a Multiparent Advanced Generation Intercross Population With a Greatly Reduced Mating Design for Genetic Studies in Winter Wheat
Source: Front Plant Sci. 2018 Dec 6;9:1825. doi: 10.3389/fpls.2018.01825 (PMC6291512; doi:10.3389/fpls.2018.01825)
Supplement: Supplementary file 7 [file Data_Sheet_7.PDF]

Chromosome 1

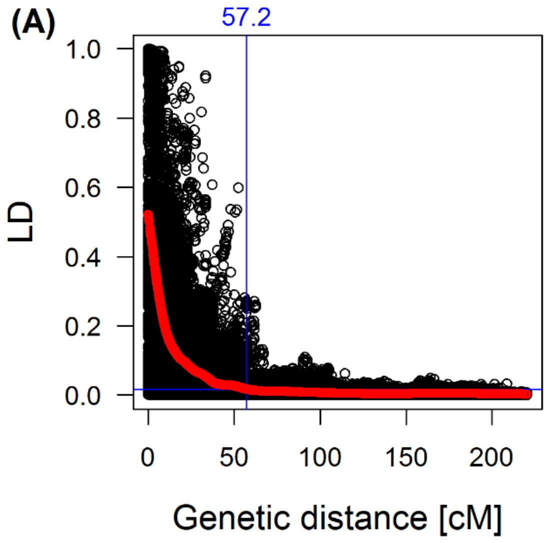

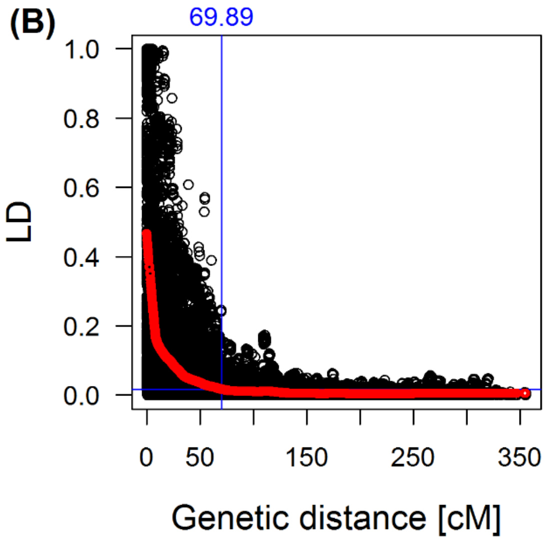

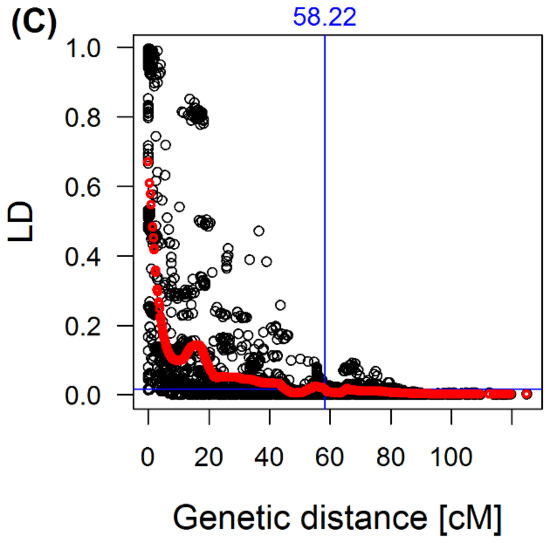

Chromosome 2

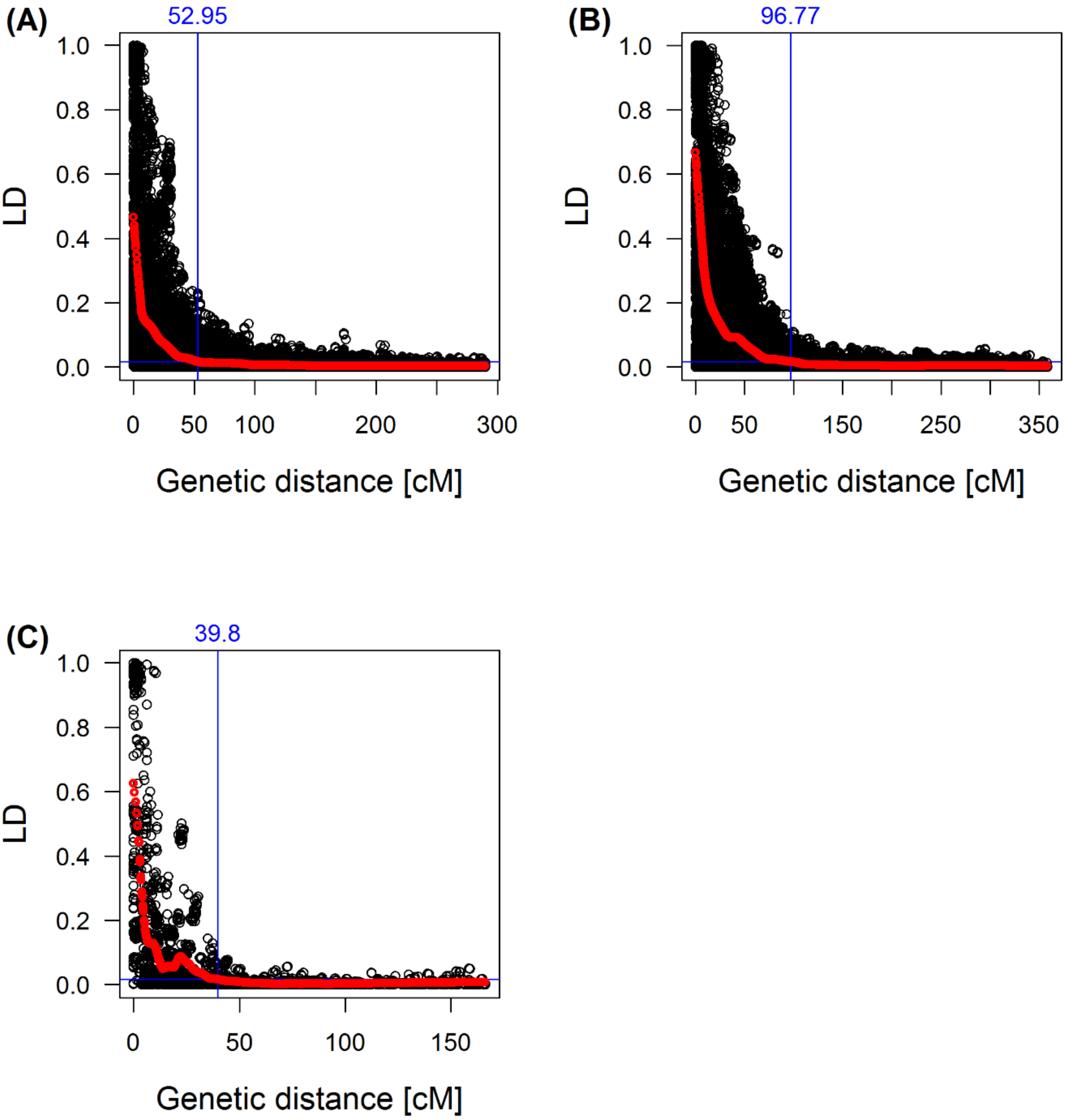

Chromosome 3

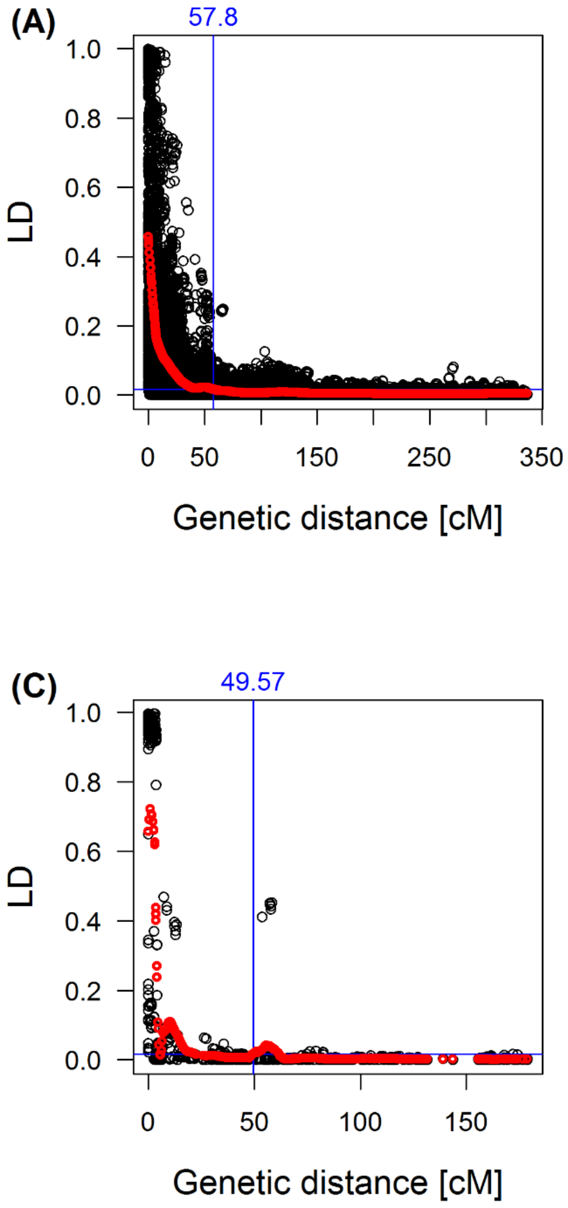

Chromosome 4

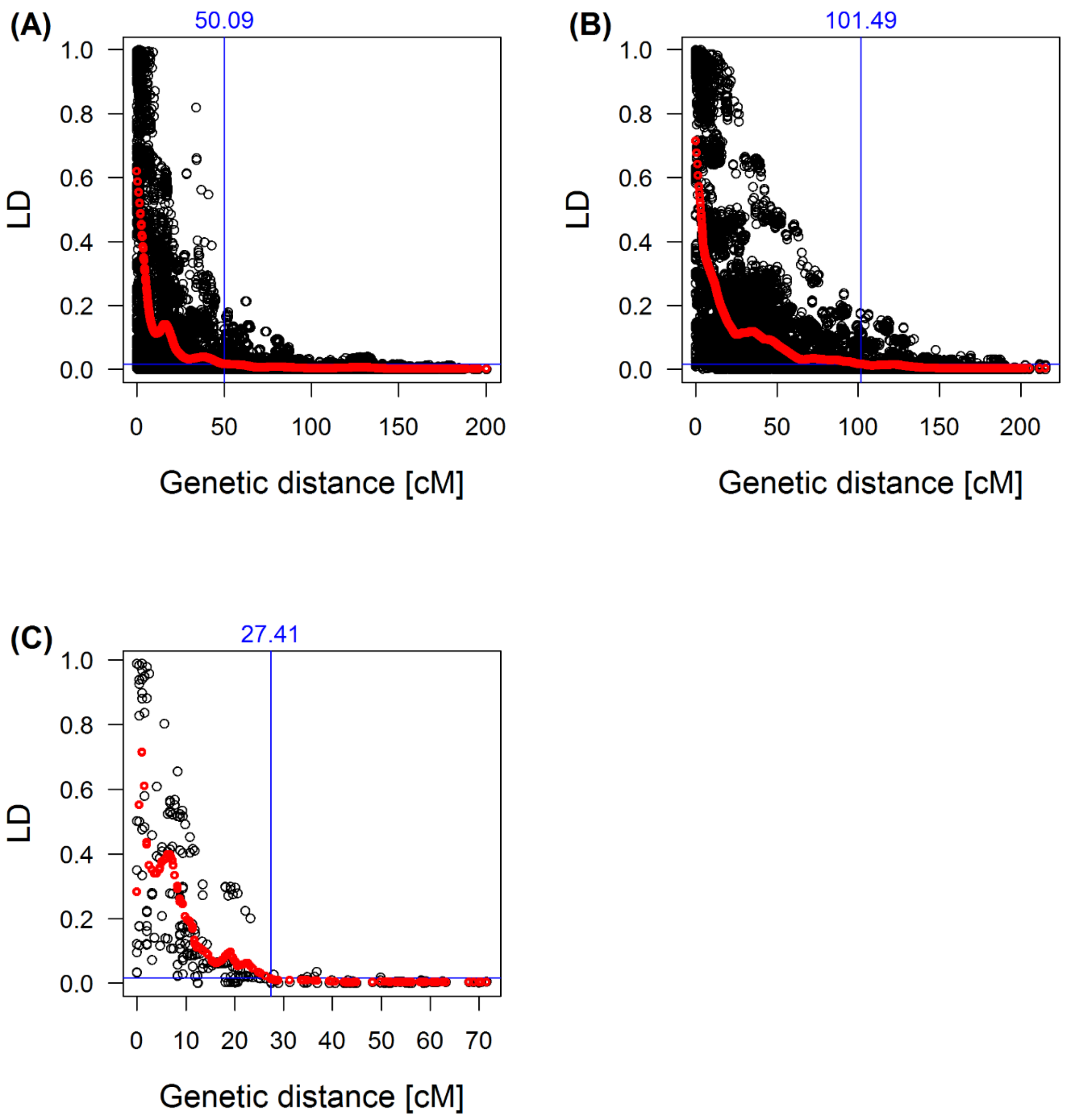

Chromosome 5

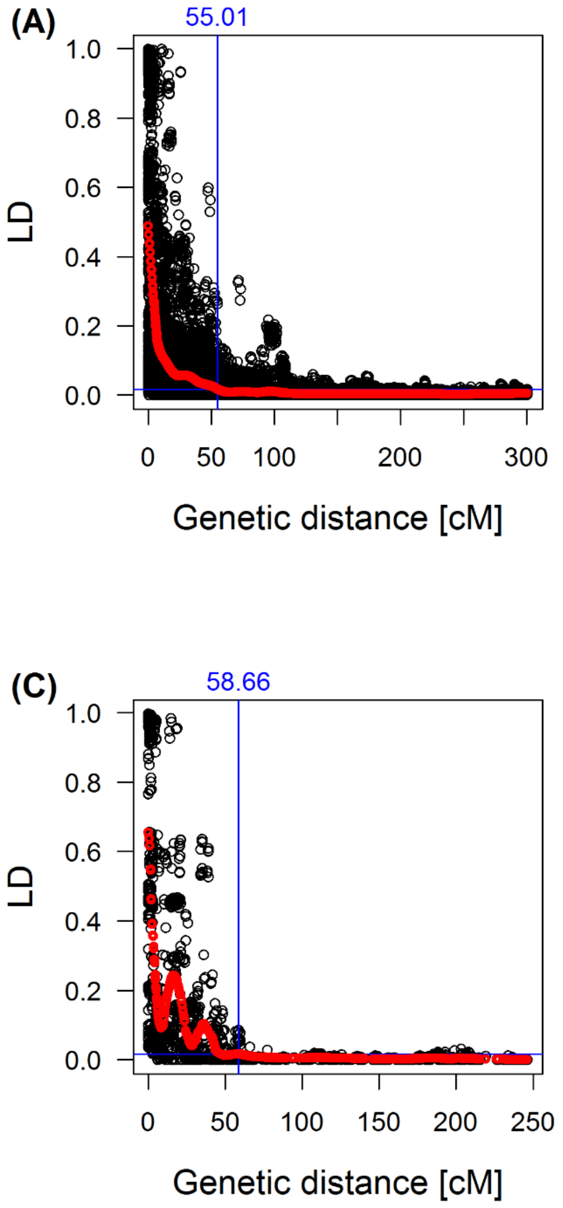

Chromosome 6

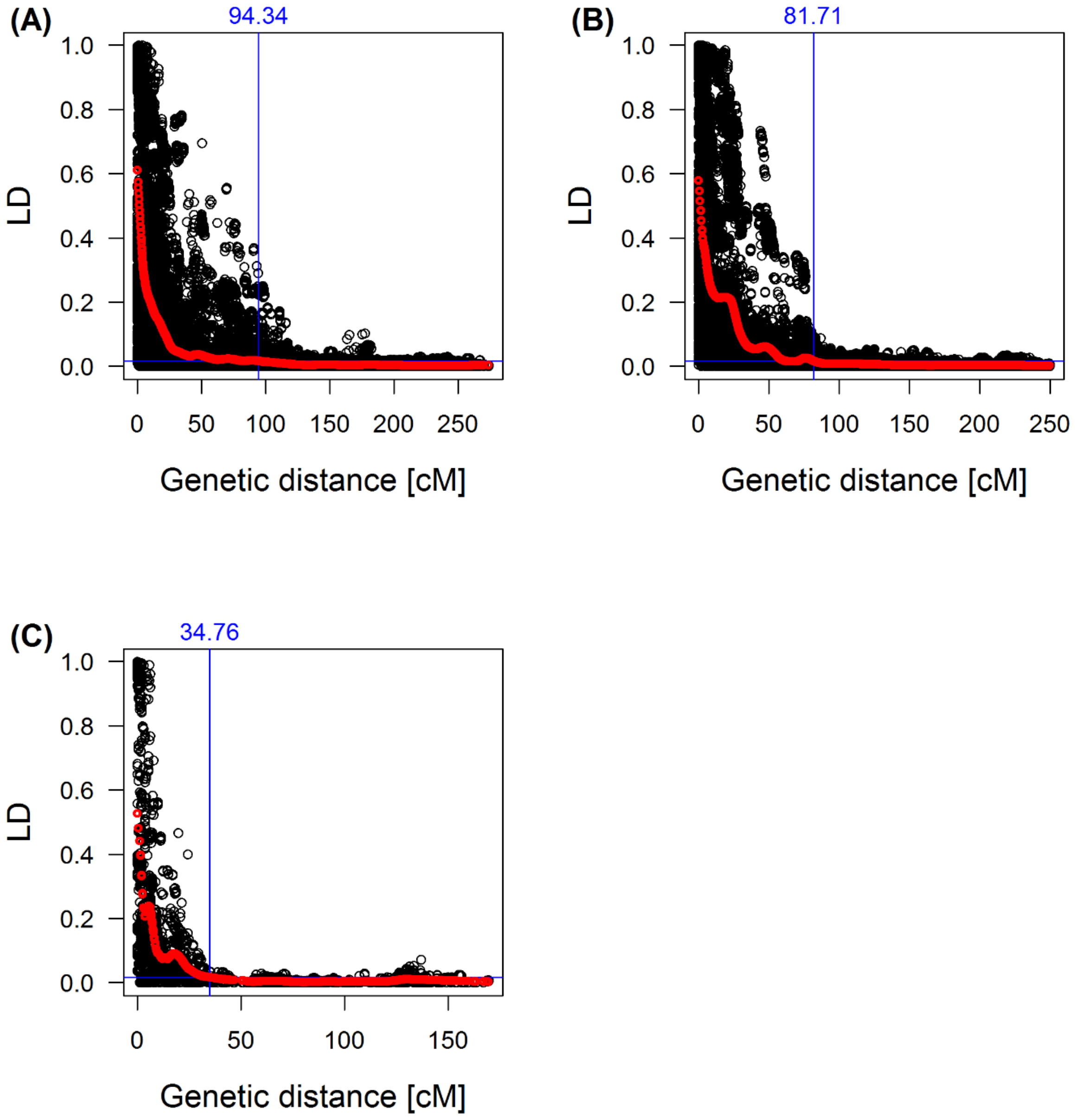

Chromosome 7

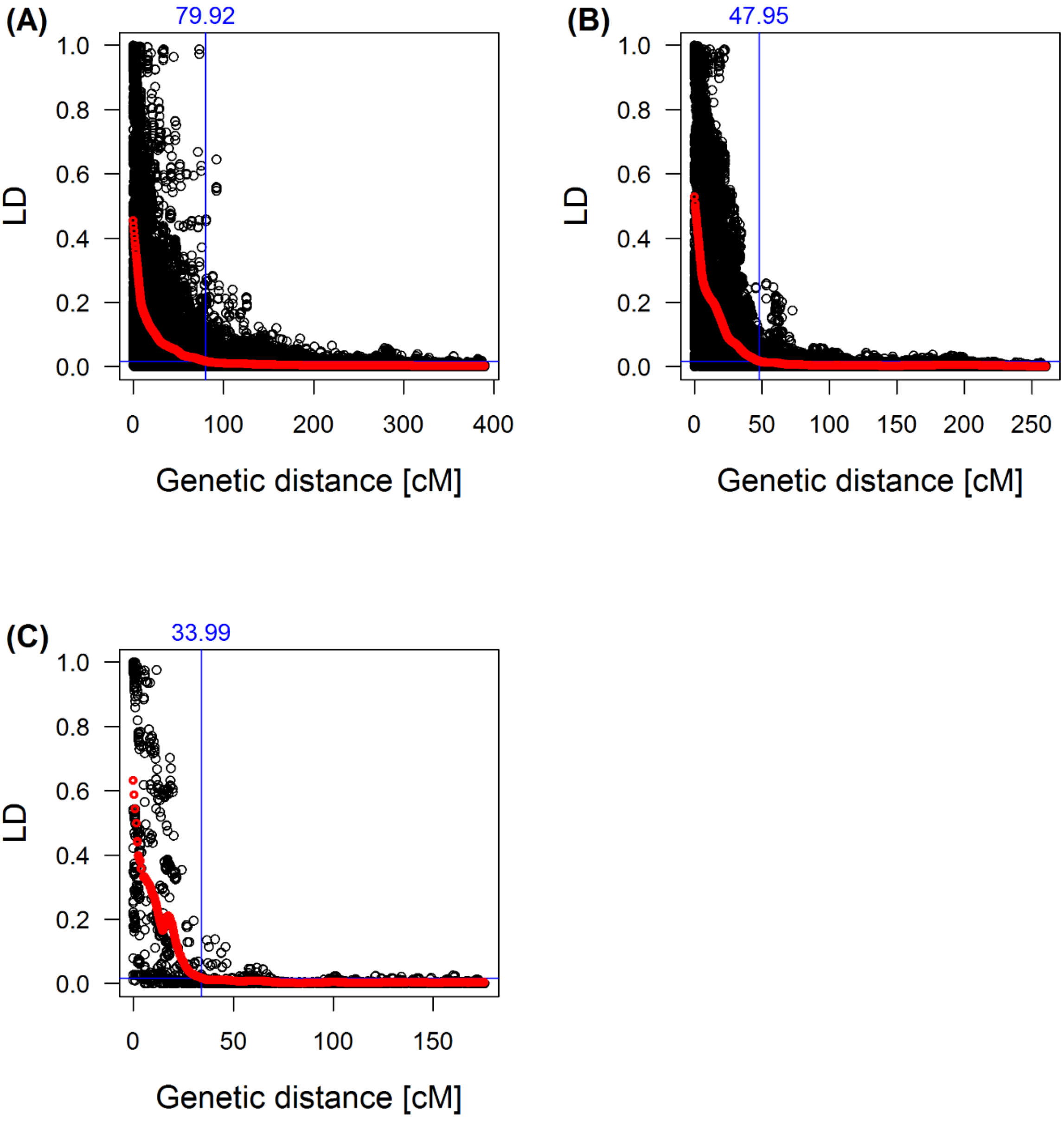

Figure S7: Linkage disequilibrium (LD) decay. (A), (B), and (C) show LD (r^2) decay of individual chromosomes from A, B, and D genome, respectively. Horizontal blue line indicates the population-specific critical r^2 value (0.017). Vertical blue line indicates genetic distance where LD decreased to < 0.017. Red line marks the fitted loess curve.
