# Supplementary material for: Usefulness of a Multiparent Advanced Generation Intercross Population With a Greatly Reduced Mating Design for Genetic Studies in Winter Wheat
Source: Front Plant Sci. 2018 Dec 6;9:1825. doi: 10.3389/fpls.2018.01825 (PMC6291512; doi:10.3389/fpls.2018.01825)
Supplement: Supplementary file 8 [file Data_Sheet_8.pdf]

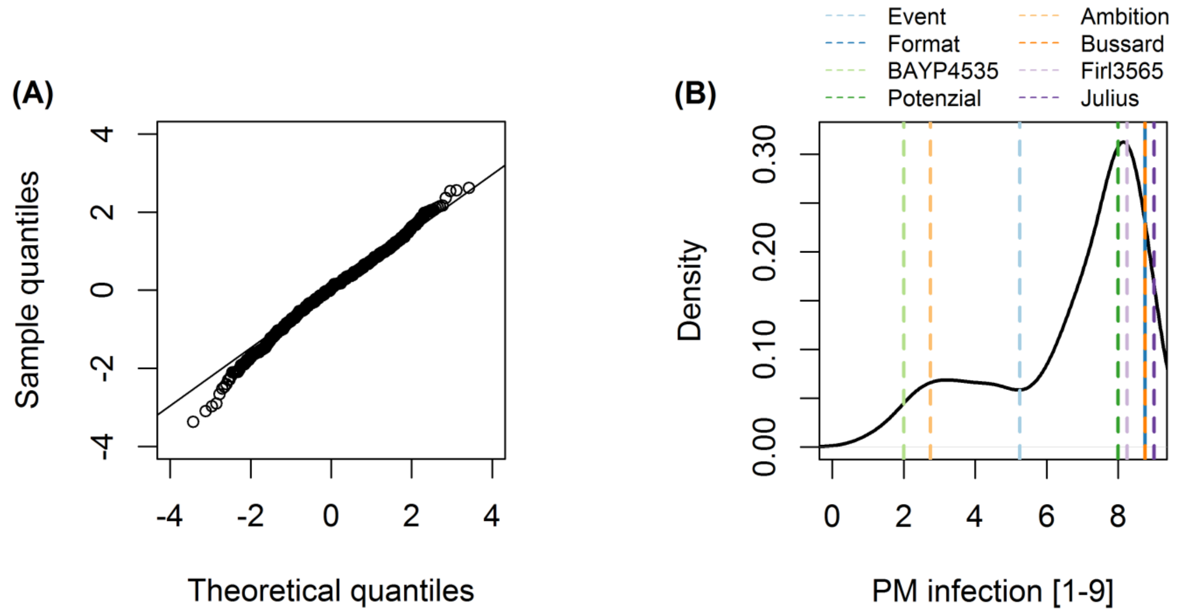

Figure S8: (A) normal QQ plot and (B) distribution of the adjusted means of powdery mildew infection. Vertical dotted lines (B) indicate the values of the parental lines.
