# Supplementary material for: Usefulness of a Multiparent Advanced Generation Intercross Population With a Greatly Reduced Mating Design for Genetic Studies in Winter Wheat
Source: Front Plant Sci. 2018 Dec 6;9:1825. doi: 10.3389/fpls.2018.01825 (PMC6291512; doi:10.3389/fpls.2018.01825)
Supplement: Supplementary file 10 [file Table_2.DOCX]

**Table S2:** Estimates of the repeatability (%), the phenotypic correlation between trials (Cor.), the mean (± standard error) of the population, the range of the population, the mean (± standard error) of the founders, the genetic, genotype * trial interaction, and residual variance component ($\hat{\sigma}_{g}^{2}$, $\hat{\sigma}_{gl}^{2}$,$\hat{\sigma}_{e}^{2}$), and the heritability ($\hat{h}^{2}$) of seedling resistance to powdery mildew (PM, score 1-9).

| Trait | Repeatability | Cor. | Mean(pop) | Range(pop) | Mean(found) | ${\hat{\boldsymbol{\sigma}}}_{\boldsymbol{g}}^{\boldsymbol{2}}$ | ${\hat{\boldsymbol{\sigma}}}_{\boldsymbol{gl}}^{\boldsymbol{2}}$ | ${\hat{\boldsymbol{\sigma}}}_{\boldsymbol{e}}^{\boldsymbol{2}}$ | ${\hat{\boldsymbol{h}}}^{\boldsymbol{2}}$ |
| --- | --- | --- | --- | --- | --- | --- | --- | --- | --- |
| PM [1-9] | 81.3, 81.9 | 0.8* | 6.7 ± 0.1 | 1 - 9 | 6.6 ± 1.0 | 4.0* | 0.1* | 0.9 | 93.0 |

^*^ Significant at p < 0.01.
